# A high-density, multi-parental SNP genetic map on apple validates a new mapping approach for outcrossing species

**DOI:** 10.1038/hortres.2016.57

**Published:** 2016-11-23

**Authors:** Erica A Di Pierro, Luca Gianfranceschi, Mario Di Guardo, Herma JJ Koehorst-van Putten, Johannes W Kruisselbrink, Sara Longhi, Michela Troggio, Luca Bianco, Hélène Muranty, Giulia Pagliarani, Stefano Tartarini, Thomas Letschka, Lidia Lozano Luis, Larisa Garkava-Gustavsson, Diego Micheletti, Marco CAM Bink, Roeland E Voorrips, Ebrahimi Aziz, Riccardo Velasco, François Laurens, W Eric van de Weg

**Affiliations:** 1 Department of Biosciences, University of Milan, Milan 20133, Italy; 2 Plant Breeding, Wageningen University and Research, Wageningen 6700AJ, The Netherlands; 3 Research and Innovation Centre, Edmund Mach Foundation, San Michele all’Adige 38010, Italy; 4 Biometris, Wageningen University and Research, Wageningen 6700AA, The Netherlands; 5 IRHS, INRA, AGROCAMPUS-Ouest, Université d’Angers, SFR 4207 QUASAV, Beaucouzé 49071, France; 6 Department of Agricultural Sciences, University of Bologna, Bologna 40127, Italy; 7 Department of Molecular Biology, Laimburg Research Centre for Agriculture and Forestry, Ora 39040, Italy; 8 Department of Plant Breeding, Swedish University of Agricultural Sciences, Alnarp 23053, Sweden

## Abstract

Quantitative trait loci (QTL) mapping approaches rely on the correct ordering of molecular markers along the chromosomes, which can be obtained from genetic linkage maps or a reference genome sequence. For apple (*Malus domestica* Borkh), the genome sequence v1 and v2 could not meet this need; therefore, a novel approach was devised to develop a dense genetic linkage map, providing the most reliable marker-loci order for the highest possible number of markers. The approach was based on four strategies: (i) the use of multiple full-sib families, (ii) the reduction of missing information through the use of HaploBlocks and alternative calling procedures for single-nucleotide polymorphism (SNP) markers, (iii) the construction of a single backcross-type data set including all families, and (iv) a two-step map generation procedure based on the sequential inclusion of markers. The map comprises 15 417 SNP markers, clustered in 3 K HaploBlock markers spanning 1 267 cM, with an average distance between adjacent markers of 0.37 cM and a maximum distance of 3.29 cM. Moreover, chromosome 5 was oriented according to its homoeologous chromosome 10. This map was useful to improve the apple genome sequence, design the Axiom Apple 480 K SNP array and perform multifamily-based QTL studies. Its collinearity with the genome sequences v1 and v3 are reported. To our knowledge, this is the shortest published SNP map in apple, while including the largest number of markers, families and individuals. This result validates our methodology, proving its value for the construction of integrated linkage maps for any outbreeding species.

## Introduction

Genetic linkage maps play a major role in clarifying the genetic control of important traits and the development of DNA-based diagnostic tools for marker-assisted breeding. They are supposed to reflect the order of genes and molecular markers as they occur on the chromosomes and are critical resources for: (i) the identification of gene location on chromosomes via quantitative trait loci (QTL) discovery studies,^1–3^ (ii) the building of reference genome sequences through anchoring, ordering and orienting of contigs and scaffolds,^4^ and (iii) the cloning of genes through map-based approaches.^5–7^ Most of the economically important traits in plant breeding, such as yield and product quality, are quantitative and controlled by multiple genes. Therefore, identifying the genomic location of such genes is a high priority for selecting new improved crop varieties.^8,9^ Remarkable advances have been achieved in understanding the functional complexity underpinning quantitative traits. A number of QTL with strong effects on phenotypic variation have been discovered, genetically positioned, validated and, in various cases, successfully exploited in marker-assisted breeding.^9–11^

In outbreeding species, conventional QTL discovery approaches rely on the availability of genetic linkage maps and segregating bi-parental full-sib (FS) families. However, a single FS family is unlikely to segregate for all QTL, thus providing only partial information. Currently, QTL mapping is shifting toward the simultaneous analysis of more complex pedigreed FS-families, derived by multiple direct parents and founders.^12–18^ This approach increases the probability of detecting QTL and capturing allelic variation while it improves the characterization of QTL performance in different genetic backgrounds.^12,19–21^

The EU-funded FruitBreedomics project^10^ was aimed, among other objectives, to clarify the genetic determination of a series of fruit quality traits in apple through a multifamily QTL mapping approach using molecular markers from a 20 K Infinium SNP array.^22^ This raised the need for a reference genetic linkage map allowing adequate integration of SNP marker data across wide germplasm. The accurateness of marker order is crucial to remove sources of spurious double recombinants and to narrow the intervals where QTL are located. When a high-quality consensus map or reference genome sequence is available, they can be used for the correct ordering of markers.

At the onset of this work, various genetic linkage maps were available for apple with most based on a single FS family^2,23–32^ and some based on a few FS families.^33–35^ Furthermore, a draft apple reference genome sequence was available,^36^ which has been used for developing whole-genome genotyping (WGG) assays^22,36–38^ for producing high-density SNP linkage maps on segregating FS families.^28,34,35^ However, all of the array and Genotyping By Sequencing (GBS) derived genetic linkage maps highlighted discrepancies in marker positions to the reference genome for ~14 (ref. 28) to 22% (ref. 32) of the markers. The generation of a highly reliable integrated map was prompted by the inconsistencies among these maps and by their low proportion of common markers.

Genetic linkage maps are created through the study of co-segregation patterns of markers and genes in segregating families. In outbreeding species, usually both parents can contribute segregation information and the generation of three different linkage maps is allowed: two parent specific maps and one integrated bi-parental map. A relevant issue in the construction of integrated genetic maps on bi-allelic markers, such as SNPs, is the high proportion of non-informative data, which are due to three main causes. First, missing values are inevitably high as most of the SNPs segregate in only one parent, thus being homozygous and not informative for the second parent. Second, markers segregating in both parents (*ab*×*ab*→*aa, ab, bb*) yield only 50% of informative data since the alleles of the *ab* progeny genotypes cannot be unequivocally traced back to the donor parent. The reduction in information is even worse when a null allele is present in both parental genotypes (*a−*×*a−*): since their progeny is called by the presence (*a*− and *aa*) and absence (−−), 75% of the genotypes (*a−* and *aa*) cannot be unequivocally called and will be uninformative. Third, most markers usually do not segregate in each family. Therefore, the total amount of missing information goes well beyond 50% for any SNP marker. Uncertainty in marker order may also arise from standard approaches for map integration when merging the two parental maps of a FS family into a single bi-parental map through automated procedures.^39,40^ This process raises ambiguity in the appropriateness of marker order due to incompleteness in segregation information. Accordingly, linkage map integration across multiple bi-parental maps further increases ambiguities due to rise in missing data.

The main purpose of the present work was to produce a highly reliable and high-density integrated multi-parent genetic linkage map for apple (*Malus domestica* Borkh) to be used as a reference genetic map and as support in improving the apple genome assembly. To obtain the most reliable order for the highest possible number of markers, a novel mapping procedure was adopted by combining the following four main strategies: (i) using 21 segregating FS-families genotyped with the recent 20 K Infinium SNP array;^22^ (ii) reducing the proportion of non-informative data through an *ad hoc* SNP filtering and calling method and by the use of the HaploBlock (HB) *bins* formed by tightly linked markers; (iii) using a backcross (BC) design on single-parent data, rather than a Cross-Pollinator (CP) design on bi-parental ones, to facilitate the integration of parental data (full details are explained in the methods section); and (iv) using a two-step mapping procedure where an Initial Framework Map (IFM) of only the most informative markers, provides a reliable starting point for adding the remaining less informative.

## Materials and methods

### Plant material

This study included 1586 progeny plants from 21 FS* *families.^22^ They were obtained from 26 parents and originated from six different breeding programs from five European countries (Table 1). Eighteen of them were part of the previous European project HiDRAS.^41^ Although most of the families comprised ~50 individuals, 7 of them significantly differed in size (Table 1), with 12_J being the smallest (23 individuals) and DLO.12 the largest (219 individuals). Part of these populations have also been used in studies on the development of multiplexes of SSR markers,^42^ validation of the pedigree-based analyses approach for QTL mapping on multiple pedigreed families^13^ and QTL discovery for horticultural traits.^18^

### DNA isolation

For each individual, young, preferably unfolded leaves were sampled and freeze dried. Genomic DNA was extracted according to Schouten *et al.*^27^ The DNA was further purified using an RNAse step and quantified using 0.8% agarose gels and a dilution series of an external reference Lambda DNA (Invitrogen, Carlsbad, CA, USA).

### SNP-genotyping

The 21 FS families and their parents were genotyped with the 20 K Infinium SNP array at the Fondazione Edmund Mach according to published procedures.^28,37^ SNP calling was performed using GenomeStudio software (Illumina Inc., San Diego, CA, USA; http://www.illumina.com), with a GenCall threshold of 0.15, and ASSIsT,^43^ a filtering and calling pipeline that accounts for the presence of null-alleles and signal intensity differences among *AB*-genotypes, thus increasing the number of usable SNP markers and providing some fully informative bi-parental markers with segregation type *ab*×*ac*.

### SNP markers origin and focal point (FP) design

The 20 K Infinium SNP array consists of three different SNP sources: the recently designed FruitBreedomics (FB) markers^22^ and subsets of the RosBREED SNPs and GD_snp_ markers (jointly referred to as IRSC—International Rosaceae SNP Consortium-markers) present on the previous 8 K Infinium SNP array.^22,37^ The GD_snp_ markers are based on polymorphisms within Golden Delicious sequence data, which were previously validated using SNPlex technology.^36^ The new FB markers were designed in clusters of up to 11 SNPs located within a region of at most 10 kbp, defined as Focal Point (FP) and distributed along the genome at distances of ~1 cM.^22^ The 8 K Infinium SNP array also followed an FP design for the IRSC markers; however, here each FP stretched up to 100 kbp.^22,37^

### Construction of bi-parental single-family linkage maps and SNP validation

Before generating the integrated multi-parent linkage map, integrated bi-parental SNP linkage maps were created for each of the 21 FS-families to validate filtered SNPs and to verify the tight linkage between SNP markers coming from the same FP. For the construction of genetic linkage maps, JoinMap v4.1 (ref. 44) was used, applying the multipoint maximum likelihood mapping algorithm approach for cross pollinators^40,45^ and the Haldane mapping function using pre-set default settings. Markers were removed from the data set of an individual FS-family when they showed a severely distorted segregation (*P*<0.01) and nearby markers segregating for the same parent did not show such a distortion, or when the rare genotype class occurred in less than 5% of the progeny. The GenomeStudio cluster plots were examined for the following: (i) markers mapping far (>10 cM) from any other marker; (ii) markers showing high nearest neighbor stress (NN stress) values (>2 cM) according to JoinMap output, and (iii) genotype calls involved in double recombinant single-points (singletons) as reported by JoinMap. When necessary and feasible, calls or the parental origin of markers were adjusted. Markers that remained problematic were excluded. Markers with identical genotypic scores (identicals), which are automatically set aside by JoinMap, were added back to the resulting linkage maps.

SNP markers from the same probe that mapped on different LGs in distinct families were classified as multi-locus SNPs, and they were considered as distinct markers specifying the mapping LG in their name. Also, the ‘$’ symbol is added in front of their initial name to easily distinguish them from single-locus SNPs. Moreover, whenever SNP markers from the same FP were mapping to distinct genetic regions, the FP was split into two or more distinct SNP clusters, or in cases of an individual SNP, this was moved out of the FP. Finally, the assessed sets of co-segregating SNP markers belonging to the same FP were defined as HBs.

### SNP assignment to HBs

Validated SNPs, as mapped in at least one of the 21 FS families (Supplementary Table S1), were grouped into HBs according to the FP design on the 20 K array^22,37^ or to their coordinates on the reference genome sequence v2 (in case of IRSC markers)^22^ while accounting for their co-segregation patterns in the individual FS families. HBs comprising only FB markers (FB-HBs) spanned at most 10 kbp, those including only IRSC markers (IRSC-HBs) covered at most 100 kbp in size, and a window of 20 kbp was assigned to HBs that comprised both FB and IRSC markers (FB+IRSC-HBs). Those SNPs that did not fall in any physical distance range allowed by the FP design were set aside the HBs and kept as individual SNP markers. Within each HB, SNP markers were ordered according to the coordinates of the targeted sequence polymorphism on the above mentioned genome sequence.

### The HB marker and the BC strategy

**Figure 1:**
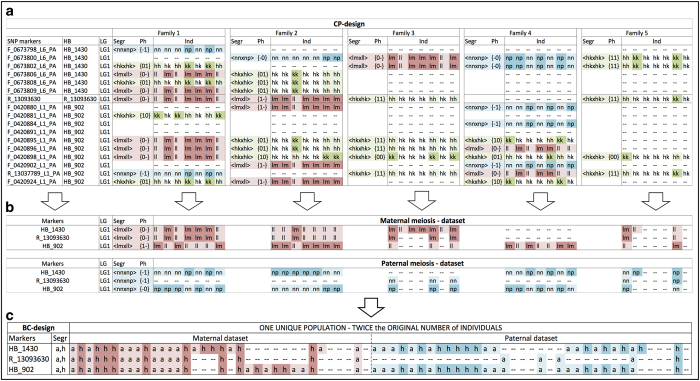
Graphical visualization of the combined HaploBlock and backcross approach presented in the current study. The figure illustrates the main steps of the process with an example from the true data of five families, each represented by seven individuals, two HaploBlocks (HBs) and one individual SNP on linkage group 1. Genotype codes presented here follow the format of JoinMap v3 and later versions for the cross-pollinated (CP) segregation types (Segr), where <lmxll> refers to a maternal marker with genotypes lm and ll, <nnxnp> to a paternal marker with genotypes nn and np, and <hkxhk> refers to a bi-parental marker with genotypes hh, hk and kk (see https://www.kyazma.nl/docs/JM4manual.pdf—Table 4). These three segregation types are highlighted with different colors: red for markers segregating only in the mother, blue for markers segregating only in the father, and green for those segregating in both parents; missing data (−−) and initially non-informative codes (hk) are not highlighted. (**a**) The use of the HB strategy allowed the identification of stable sets of SNP-markers, such as those composing HB_1430 and HB_902 that consist of 6 and 10 SNPs, respectively. These SNPs do not segregate in all families (the only exception is F_0420898_L1_PA), thus leading to a considerable amount of missing information (62% of data points). (**b**) The genotypic information of the co-segregating SNPs is aggregated to form a single HB marker across families and the bi-parental allelic contribution is also split to form two distinct single-parent data sets, where the phase of the new ‘single parent’ HB-markers is adjusted accordingly. (**c**) The two complete single-parent data sets are subsequently converted in a backcross (BC) design and combined to form a unique population of twice the number of individuals as the initial CP populations. The presented strategy permits the almost complete exploitation of the segregation information available (losing only some information from the rare recombination events within a HB) while considerably reducing the amount of missing information: in this example, from 76% for the initial CP data sets of the two HBs to 28% in the final unique BC population. For the single SNP, the amount of missing data did not change throughout the process by definition and was 66%. This approach of data aggregation and mating type was implemented in the software Haplotype Aggregator (HapAg—http://www.wageningenur.nl/en/show/HaploblockAggregator.htm), whose manual describes the process in more detail.

The creation of HBs of co-segregating markers allowed a *bin-*mapping strategy where the segregation information of adjacent SNPs was aggregated and condensed into a single, virtual HB marker. The aggregation of co-segregating markers within the same HB increases the genotype score robustness consequently to information redundancy, and marker informativeness when combining markers with different segregation types. This is the case when a marker segregating in a single parent (for example, *ab*×*aa*) is combined with a bi-allelic marker heterozygous in both parental plants (for example, *ab*×*ab*) or with a single parent marker of the other sex (for example, *aa*×*ab*), leading to the generation of a fully informative marker record (corresponding to a segregation type *ab*×*ac*, or *ab*×*cd*). In view of our mapping effort, this strategy was implemented in the *ad hoc* developed software Haploblock Aggregator (HapAg—http://www.wageningenur.nl/en/show/HaploblockAggregator.htm) and applied to our data (Supplementary File S1). For each FS family, HapAg aggregated the segregation information of the SNP markers belonging to the same HB by using the information on linkage group and the linkage phase of the individual markers (Figure 1a), while considering the meiotic events occurring in the two parental plants separately. Thereto, HapAg splits the parental allelic contribution of every individual of each family into two distinct sub-data sets including either maternal or paternal recombination events (Figure 1b). Eventually, maternal and paternal data sets of all the progenies from all FS-families were merged to generate a single BC-type data set (Figure 1c), having twice the number of individuals as the original CP populations (a more detailed description of the methodological steps performed by HapAg is available in the software manual (http://www.wageningenur.nl/en/show/HaploblockAggregator.htm)). The BC segregation type allows the correct phasing of the markers segregating in different families, leading to integrating the genotypic data prior to map construction and to the production of a unique integrated genetic map rather than a map resulting from the *a posteriori* integration of the linkage maps obtained from FS families.

When inconsistencies between SNP scoring within the same HB are present (for example, due to recombination or gene conversion within the HB or calling issues), warning messages are reported, and the aggregated data score is set to missing (−−) by HapAg. The warnings were carefully examined to identify the origin of the issue and, when possible, to solve it before producing the final data set. Specifically, when conflicts occurred in more than five individuals per HB across all families, these were always inspected, while less consideration was given to a lower number of warnings as compatible to the expected number of true double recombination. Given the overall estimated genetic to physical size ratio of 548 kbp/cM, based on the results from Velasco and colleagues,^36^ the probability of a recombination event occurring within a single HB is expected to be 1.8×10^−4^ (FB−HBs), 1.8×10^−3^ (IRSC−HBs) and 3.5×10^−4^ (FB+IRSC−HBs). These probabilities are so low that we decided to neglect such recombination, at least during the construction of the map, and thus, make missing the HB marker call for the potentially recombinant individual.

The final BC-type data set, formed by HB markers and individual SNPs not included in HBs (for simplicity, the generic term ‘markers’ will be used to refer to both), was used to construct the integrated Genetic Linkage Map (iGLMap).

### iGLMap construction

Linkage maps were produced using JoinMap v4.1 with the same algorithm and parameter settings used when mapping the single FS-families, but now following a BC design. Due to the sensitivity of mapping algorithms to missing data,^40^ a two-step mapping procedure was adopted. First, a reliable IFM was determined by including only markers that had genotypic data for at least 25% of the individuals (from 800 to ~3 200 available meiosis). Subsequently, the map was completed by adding less informative markers with up to 90% missing data (from 320 to 800 available meiosis), while using the obtained IFM marker order as the Start Order in JoinMap. Markers with more than 90% missing values (327) were not considered. At both steps, for each linkage map, double recombinant single points (singletons) identified by the JoinMap Genotype probabilities function were visually examined through a graphical genotyping approach^46^ whereby data were displayed in map order as color-coded genotypes in Microsoft Excel using the conditional cell formatting feature. Issues causing singletons were investigated. For each LG, the best map was defined as the one with the lowest number of singletons.

### The iGLMap for apple genome improvement

The produced IFM was used to examine and improve the reference apple genome sequence from v2, as used for the design of the 20 K Infinium SNP array,^22^ to v3 (https://www.rosaceae.org/species/malus/malus_x_domestica/genome_v3.0.a1). Specifically, this map helped to assess potential issues in the anchoring of contigs within scaffolds and provided a new assignment of scaffolds to chromosomes.

To confirm the collinearity between v3 of the reference genome and the final iGLMap, physical coordinates of all genetically mapped SNP markers were plotted as a function of genetic distances on the iGLMap. For comparison, collinearity between the iGLMap and the most widely used apple genome sequence v1 was also evaluated. MareyMaps^47^ for each chromosome were produced using R (R Development Team Core, Vienna, Austria, Europe).

## Results

### Construction of 21 bi-parental linkage maps

The 21 FS families (Table 1) were genotyped using the 20 K Infinium SNP array.^22^ Overall, 15 697 SNP markers (87%) passed the SNP calling and filtering pipeline ASSIsT^43^ and were genetically mapped using a final set of 1 586 individuals. Single-parent map integration produced high-quality bi-parental maps for each family and LG (Table 2; Supplementary File S2), with six exceptions. LG6 and LG16 in family I_W and LG7 in family I_CC show large homozygous regions which led either to the absence of segregating bi-parental bridge markers (thus only single parent maps could be generated) (Supplementary Figures S1a and b), or to the lack of segregating markers in one parent (LG7 I_CC). Furthermore, LG17 in family I_CC has no integrated map because the few bi-parental markers are not sufficiently spaced to allow orientation of the two single parent maps. This family is also peculiar since its paternal LG17 shows a highly distorted segregating region, coincident with the self-incompatibility *S* locus (Supplementary Figure S1c), thus suggesting the two parents to have a common self-incompatibility allele. Finally, the LGs 13 in families 12_J and 12_K have a genetic length of 1.5–1.6x the population mean (Table 2). Such relative large sizes are usually an indicator for data issues. This was true for 12_J, where the family size showed to be too small to come for a meaningful genetic map for this LG. For 12_K however we suspect a biological reason as its distorted segregations and double recombination pattern may be explained by the presence of natural selection at distant loci of the same chromosome (data not shown).

The total length of the 21 maps ranges from 1123 (GaPi) to 1 551 cM (12_K) with an average length of 1 305 cM (±113.3) across families (Table 2a). The average distance between SNPs ranges from 0.16 cM in JoPr to 0.25 in 12_K. On average across families, 6 848 (±619) SNPs were mapped ranging from 5 570 (12_N) to 8 454 (JoPr) (Table 2b).

### Focal points validation and HBs identification

The order of markers on the 21 genetic maps was thoroughly checked, and correspondence to the apple’s physical map v2 was assessed. Overall, the SNP marker order was coherent. Nonetheless, the following three main issues were identified that were common to all families and, overall, affected 22% of the markers: (i) inconsistencies in LG assignment for 17% of the markers; (ii) regions of inversion (max ~2.5 Mbp) involving 3% of the markers; and (iii) misplaced regions of markers within the same pseudo-chromosome for 2% of the markers. Furthermore, a close inspection of the bi-parental genetic maps highlighted the presence of 111 multi-locus SNPs (0.7% of the markers), mapping in 2 or even 3 (on 4 occasions) different LGs across distinct families (Supplementary Table S2); 29 of these fall in known homoeologous regions originating from an ancient apple genome duplication.^36^ The 111 multi-locus SNP probes resulted in additional 115 alternative mapped SNP loci, establishing a final total number of 15 812 validated SNP-loci, each segregating in at least one family (Table 3, Supplementary Table S1).

Therefore, the expected tight linkage between SNP markers of the same FP could not always be confirmed because 91 FPs included SNPs mapping to distinct genetic regions. Such discrepancies led to the creation of independent SNPs sub-clusters, identified as distinct HBs or individual SNPs. At this stage, 921 individual SNP markers and 2 837 HBs were considered (Table 4).

### HB marker genotype definition and BC data set

The software HapAg was designed in the framework of the current mapping effort to aggregate the segregation information of the SNPs belonging to each HB into a single HB marker. During the aggregation process, HapAg reported 2 848 inconsistencies in SNPs scoring within 748 HBs (26%). More than half of them (51%) resulted from the erroneous assignment of 55 SNPs to a FP and, therefore, to a HB, due to inadequate genome sequence information (unidentified during the mapping effort on single families). Those SNPs were removed from the HBs and used as individual SNP markers (Supplementary Table S1). The remaining 49% of conflicts involved 25% of the HBs and were due to calling errors, recombination within HBs, or gene conversion.

The majority of these HBs (96.3%) presented no more than 5 warnings, which were considered having a minor impact. The remaining 3.7% reported between 6 and 16 conflicts. These inconsistencies were addressed by setting the HB score of the conflicting individual as missing. In fact, it would have been unfeasible to examine all cluster plots discriminating between calling errors, true recombination events, or gene conversion in a reasonable amount of time.

**Figure 2:**
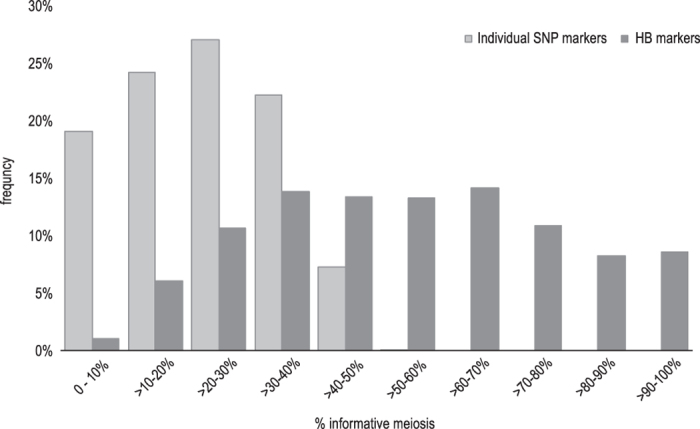
Frequency distribution of informative meiosis (in percentage) in the initial set of individual SNP markers (light gray bars) and the final set of HaploBlock (HB) together with remaining individual SNP markers (dark gray bars). The graph highlights the different amount of informative meiosis carried by individual SNPs and the more informative HB markers. SNP markers carried a maximum of 50% of the total information when being completely bi-allelic, as expected, and a maximum of 60% when being tri-allelic in some families when accounting for null-alleles and signal intensity differences. However, the latter is true only for a small proportion (0.1%) of the SNPs, while the majority of SNPs is informative for 20–40% of the individuals. On the contrary, the majority of HB markers (+remaining single SNP) are informative for 40–80% of the individuals across all families and even 8.6% of the HBs is fully informative (100%).

The final integrated data set consisted of a single BC-type population of 3 172 individuals, including the genotypic information of 2837 HB makers and 976 individual SNPs. The overall proportion of missing information was massively reduced from 78% of the initial set of 15 812 SNP markers to 54% of the final (HB+individual SNPs) integrated data set (Figure 2), thus retaining the complete genetic information of the larger SNP data set.

### Construction of the iGLMap

An IFM was constructed using markers that had data for at least 25% of the 3 172 individuals, which was true for 2631 HB and 344 individual SNP markers. The genetic length of its 17 LGs ranged from 64 (LG1) to 113 cM (LG15), for a total IFM length of 1 279 cM (Table 5). The IFM was subsequently completed by adding markers with genotypic data for at least 10% of the individuals, to produce the iGLMap. Over the whole mapping process, an improvement in map quality was obtained through the close inspection of 1 320 singletons by graphical genotyping (Supplementary File S3), whereby 67% of the singletons showed to result from 386 misplaced markers. These were moved to nearby positions. The adjusted marker order was then used as the Start Order for map re-estimation verifying that singletons’ issues were indeed solved, and no new double recombinants were generated by the shift. Next, 13% of the singletons came from 13 HB markers that could not be adequately placed along the map, because they showed conflicting genotypes with adjacent markers at any position. Since the underlying cause could not be identified, those HB-markers (Supplementary Table S1) were removed. Finally, 20% of the initial 1 320 singletons remained. These are likely caused by genotyping errors, gene conversion events or true double recombinations. As the examination of each individual case would have been too time intensive, singletons’ scores were set to missing, following Bassil *et al*.^48^

**Figure 3:**
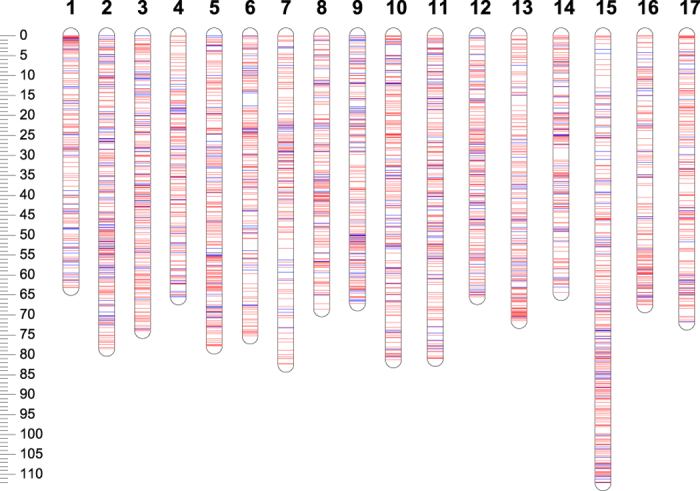
Visual presentation of the distribution of the iGLMap haploblock markers (red lines) and single SNP markers (blue lines) over the 17 linkage group apple. Position and name of the markers is detailed in Supplementary Figure S3.

The final iGLMap (Figure 3, Supplementary Figure S4) utilized 15 417 SNPs (Table 3) and consisted of 3 473 markers as follows: 2 797 HBs (Table 4), composed of a total of 14 741 SNPs, and 676 individual SNP markers (Table 5). The total genetic length is 1 267 cM, with LG1 being the shortest linkage group (63 cM) and LG15 being the longest (112 cM). The maximum distance between adjacent markers of 3.29 cM is observed on LG6 (Table 5). An estimate of marker position robustness is provided by JoinMap Plausible positions output table where, for each marker, the probability of its assigned and alternative positions are estimated across 1000 iterations. Graphical results for each LG are presented in Supplementary File S4. The order of markers appeared highly reliable, as all 1000 iterations resulted in a single position for most markers. Only a few regions show plausible alternative marker orders. Generally, such regions are characterized by very high marker density. When only two markers are involved, the distance between such markers is usually less than 0.01 cM. When three or more markers are involved, they usually span a region of less than 0.05 cM (with the only exception of four regions in total, one on each of the linkage groups LG1, LG9, LG10 and LG15, which spanned from 0.07 up to 0.1 cM). Thus, the overall results show a robust and accurate marker order along the iGLMap with just a few small regions of uncertainty where the resolution was not enough to distinguish the genetic order of tightly linked markers.

### iGLMap collinearity with the apple genome

**Figure 4:**
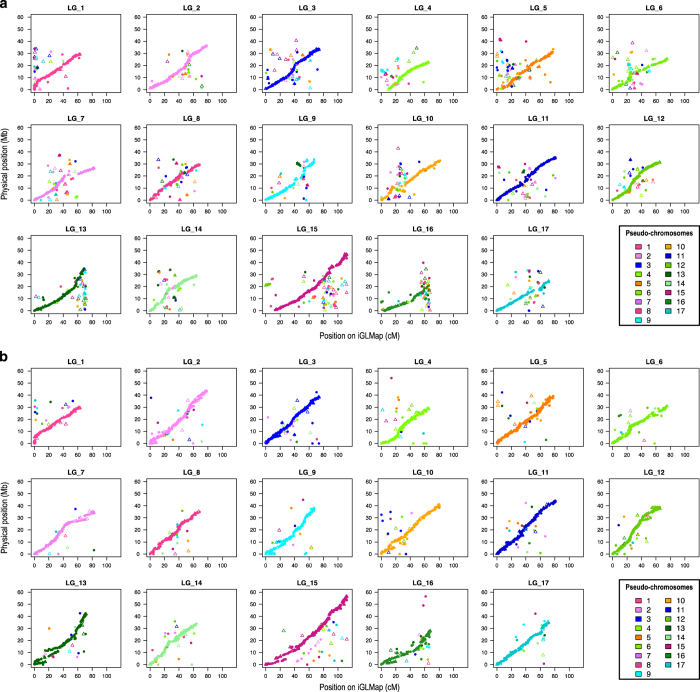
Collinearity between the integrated Genetic Linkage Map (iGLMap) and v1 (**a**) and v3 (**b**) of the apple genome sequence. Physical coordinates for SNP markers were retrieved from v1 (15 133 SNP markers traced) and v3 (14 539 SNP markers traced) of the Apple Genome and their physical positions (Mb) are plotted versus marker genetic positions (cM). Filled dots and triangles indicate markers physically positioned on the Primary Assembly sequence and alternative scaffolds sequences, respectively. Different colors indicate different pseudo-chromosomes according to the legend in the figure; thus, markers inconsistently assigned to Linkage Groups (LGs) and pseudo-chromosome are immediately highlighted.

The 21 bi-parental genetic maps already led to the identification of many regions where the genetic and physical maps deviate from collinearity. The final iGLMap allowed for visualization of inconsistencies with version v1 and v3 of the apple genome^36^ (Figure 4). The most used version is v1, which consists of a Primary Assembly and various alternative scaffold sequences (Figure 4a) representing homologous and possibly also some homoeologous sequences. Genetic data suggests the presence of some inverted regions that can be recognized by short lines at a right angle to the main curve of each graph in Figure 4a, which goes from the bottom-left to the top-right. Two examples are approximately at 20 cM on LG10 and at 35 cM on LG11. The short sequences of dots clearly aligned above or below the main curve represents regions where the position on the genome sequence may be shifted (that is, approximately at 80 cM on LG10, at 75 cM on LG11 and at 60 cM on LG14). Some regions of the iGLMap have their sequence counterparts on non-corresponding pseudo-chromosomes. For example, the initial part of LG1 and the 22–34 cM region of LG10 include markers belonging to other nine pseudo-chromosomes according to the genome sequence; also, the initial region of LG4 (0–10 cM) consists almost entirely of markers from the pseudo-chromosome 9. Alternative scaffold sequences may also combine information from multiple pseudo-chromosomes. For example, the alternative scaffold on pseudo-chromosome 9 seems to contain, for the 20 Mb region, a small fragment of its homoeologous region on LG17.

To support the development of a new 487 K Axiom array,^38^ version 3 of the apple reference genome was developed by using, among others, genetic information from the IFM. Adjustments were made only when entire contigs could be moved. Though overall collinearity has greatly improved, 2% of the genetically mapped markers is not represented anymore, and inconsistency is still observed for 6% of the markers. As shown in Figure 4b, collinearity still varies considerably as follows: some LGs need further attention for just some narrow regions (for example, LG1 and LG17), while others have several regions to be ameliorated (for example, LG13 and LG16). At least six LGs show lines parallel to the main curve, suggesting the presence of scaffold sequence segments that have been misaligned (LG7, LG9, LG13, LG14, LG15 and LG16).

## Discussion

The iGLMap was developed through an innovative mapping approach whose power and major novelties rely on the use of HBs and a single BC-type data set. The HB approach provided highly informative common markers across families, facilitating integration of the maps. The use of the BC strategy applied to an outcrossing species allowed the integration of the genotypic data prior to map construction, avoiding the multiple efforts of parental map integration within and across FS families. The advantages of the HB strategy and BC design were strengthened by the use of an improved SNP filtering tool (ASSIsT^43^), which increased the number of informative markers; and by a multi-step map construction, which facilitated the inclusion of markers with relatively high missing value content in the final high-density linkage map.

### Use of multiple FS families

The production of multifamily genetic maps can lead to an increase in marker density and genome coverage, overcoming local loss of genetic resolution and thus having the potential for a more accurate order of markers.^49^ To our knowledge, the iGLMap features the highest number of families ever used for a crop plant’s genetic mapping, followed by 13 families for wheat.^49^ Indeed, the merging of data from 21 FS families resulted in an integrated map with an average of 2.3 times the number of markers of its preceding single family maps and a genetic length highly consistent with that of the preceding maps (284 cM shorter than the longest and 144 cM longer than the shortest one). The enhanced map resolution is indicated by the consistent reduction in the maximum distance between adjacent markers as follows: 3.29 cM on the LG6 of the iGLMap, compared to values for individual families ranging from 5.94 (LG7 of the FuGa family) to 30.14 cM (LG13 of the 12_K family). The use of multiple families thus allowed for overcoming the limitations that occurred in single families including issues related to large regions of homozygosity and to extremely skewed segregation ratios, as those reported here for families I_W and I_CC, and solved in the final iGLMap.

### Use of improved SNP calling and HBs

The essence of our strategy is the reduction of missing information, which usually hampers accurate marker ordering. Therefore, we aimed to include as many common SNPs as possible by exploiting information from null-alleles, and to increase information content of bi-parental *ab*×*ab* markers also by exploiting signal intensity differences.^43,50^ This contrasts with standard SNP calling procedures, which usually do not recognize null-alleles and discard such markers while classifying them as ‘non-Mendelian’.

The use of null-alleles is expected to improve mapping efficiency not only by increasing the number of common markers, but also by increasing information content of single SNP markers: this allows the use of tri-allelic markers of type *a0*×*b0* that provide full segregation information for both parents.^43,50^ Accounting for differences in signal intensity due to an additional SNP at the probe site also gives rise to fully informative markers, because two variants may occur from one or both of the marker alleles converting bi-allelic *ab*×*ab* markers into tri-allelic *ab*×*ab’* ones.^43,50^

Our data set included 1 244 SNP markers with null-alleles that segregated in at least one FS-family, resulting in additional data for a total of 1 974 marker/family combinations. Of these, 327 markers resulted in 494 (25%) combinations that showed full segregation information (*ab*×*ac*), 514 markers out of 915 (46%) combinations that segregated as dominant *ab*×*ab’* markers for which a null-allele was present in both parents. Additionally, 328 initial *ab*×*ab* markers became fully informative *ab*×*ac* markers for 471 combinations by exploiting signal intensity differences resulting from an additional SNP at the probe site. The accounting for null-alleles and for differences in signal intensity thus increased the amount of segregation information for HB-markers and individual SNP-markers. The impact of this method on the final data set will vary with the study population and will increase at decreasing numbers of families and HB size.

The proposed HB strategy may show some similarities with *bin *mapping, although it presents substantial differences that can lead to a more accurate marker positioning. Usually, *bin* mapping is based on the identification of an interval (*bin*) along a linkage group where, given a small set of individuals, no recombination events are observed for any of them.^51^ This approach has been very successful to obtain approximate genetic information on marker position along a chromosome using a small number of individuals.^52–54^ However, the smallest unit of resolution in these genetic maps was given by the *bin*, whose length usually spanned from an average size of 7.8 cM up to a maximum length of 33 cM.^52,54^ Conversely, our HB strategy defined *bins* based on (very short) physical distances. The use of multiple SNPs within a narrow physical *bin* was anticipated in the design of the 8 and 20 K Infinium SNP arrays through the identification of tightly clustered SNP markers within a small physical window.^22,37^

Indeed, the HB strategy was very effective as it led to an increase in marker robustness through the exploitation of redundant genetic information of adjacent markers, and to an increase in information content, especially when markers were of different segregation type. In fact, in pseudo-testcross analysis,^39^ bi-allelic markers heterozygous in both parents lead to 50% non-informative data as it is not possible to unequivocally establish the origin of the alleles in the heterozygous progenies. On the contrary, the proposed HB strategy is able to exploit these data to produce a robust and fully informative HB marker when such bi-parental markers are combined with markers of other segregation type(s) within the same HB (see family 2 in Figure 1). Consequently, whereas all single bi-allelic SNP markers were informative for less than 50% of the progenies across multiple families (Figure 3), 55% of the aggregated HB-markers are informative for more than 50% of the individuals, and 8.6% of the HB-markers presented 90 to 100% of the information, thus providing highly informative bridge markers common to most FS-families. The use of HB-markers thus favored map integration across families and allowed high accuracy in marker position along the chromosome.

In this study, we did not further examine within-HB SNPs order, and we disregarded *a priori* any recombination inside HBs by making the relative marker scores missing. However, the within-HB recombinations may still be useful in the future to validate local genome assemblies when needed. We estimate that ~1 940 within HB recombinations will be present in the whole data set. This estimation is based on a genome-wide relationship between physical and genetic distances of 586 Kb/cM (the estimated genome size of 742 Mb [ref. 36] divided by the length of the iGLMap), the physical length covered by the HBs (using the number of mapped HBs and their maximum allowed size for each of the three different HB-type), the total number of individuals (3 172), the availability of informative data (46%), and assuming absence of crossover interference.

This estimated number is certainly inflated, as crossover interference does occur. Furthermore, the distance between the two most apart and informative SNPs within a HB is less than the allowed maximum HB size. Also, not all these recombinations will be noticed as having occurred within the HB, because, in many parents, HBs lack the two or more informative SNPs needed to observe recombination. Consequently, the number of expected observable recombination events might not be very different from the 1 396 conflicts observed among SNP calls within HBs. Notably, if such estimations are correct, they also imply the absence of major calling issues in the final data set.

The HB approach makes use of all the available markers, including identical markers *sensu* JoinMap. These are markers that have exactly identical genotype calls across all progenies of a single mapping family, including an identical linkage phase. In the analysis of single families JoinMap removes such identicals from the data set by default, thus reducing memory requirements and computation time. However, we re-entered them because they are needed for the construction of HB markers. The availability of full genetic information for each HB, completed by the use of the identicals, allows the adequate mapping of these HBs. Furthermore, in the case of conflicting data within a HB, the presence of identical markers may be helpful in tracing the inconsistency.

### BC mapping strategy

The most commonly used linkage analysis approach for outbreeding FS families is based on the two-way pseudo-testcross strategy,^39^ which results in the production of two single-parent maps to be then integrated using the available bi-parental bridging markers. Despite the advances in map integration methods^40,55–58^ the process is still based on computing the average of marker distances over the two parental maps, which is affected by the different segregation types and by the number and informativeness of bridging bi-parental markers. The consequent result is a loss of parental-specific features, low accuracy in marker order^4^ and inconsistencies between individual maps. Similarly, the most popular approaches used in the generation of multi-population consensus maps^59–61^ are based on the integration of marker distances over populations. Therefore, map integration is not a straightforward process, especially because usually not all the loci are common in all populations, and additional factors, such as the local reordering and marker misplacement, further affect resolution and accuracy of the consensus map.

On the contrary, the proposed BC design, leads to the generation of a single data set where genotypic data have been merged prior to the generation of integrated genetic maps. This strategy reduces the integration process to a single step where segregation data, recombination events and marker order are directly related and accessible for close inspection across all germplasm through visual approaches, such as graphical genotyping.^46^ The use of this strategy to obtain high-quality linkage maps is demonstrated by the short length of the iGLMap and the robustness of its marker order (see below). Moreover, the use of a condensed, fully informative data set optimizes computational performance.

### A multi-step mapping procedure and data curation through visual inspection

Multi-step procedures are very often used for the construction of high-density consensus maps as they allow for marker order optimization and genotyping error correction.^62^ In standard mapping approaches, bi-parental segregating framework markers can be used to produce back-bone maps, which provide a fixed order on which the final consensus map is built by adding the remaining markers.^63^ *Bin*-mapping methods also follow a two-phase mapping process based on the initial construction of high-confidence framework maps to which new markers are subsequently added.^25,51,64^

In our case, the choice to pursue a two-step mapping procedure was further motivated by the sensitivity of the JoinMap mapping algorithms to the presence of missing data.^40^ In fact, JoinMap was not able to perform the grouping when analyzing all 3473 markers simultaneously. For this reason, we first decided to produce a robust IFM including only the most informative markers.

Nevertheless, in high-density marker data sets, the marker order proposed by JoinMap demonstrated sensitivity to the presence of single problematic data points^48^ and/or structured missing data as present in our final data set due to markers segregating in some families but not in others. This may lead to inadequate marker orders that can cause inflation of map size, a high number of singletons and larger regions of double recombination. In Bassil *et al.,*^48^ as in our current study, the use of a graphical genotyping tool was crucial to perform adequate data curation through identifying problematic data points and alternative marker orders for improving the map quality and reducing singletons and double recombination.

### Evaluation of map quality

High quality and robustness of the iGLMap is inferred from its genetic length when compared to other published linkage maps of apple. Since the release of the genome sequence of apple^36^ and the consequent extensive development of whole genome genotyping SNP arrays,^22,37^ several high-density genetic maps of apple were constructed. Most of them are integrated bi-parental maps of one family, where the number of individuals ranges from 118 to 297, comprising different type of markers (for example, SNP+SSR for a total of 1,016 markers;^34^ SNP+SSR for a total of 2 579 markers;^28^ SNP+SSR for a total of 601 markers;^65^ 3 441 SNP (ref. 66)) and GBS based linkage maps comprising 3 967 SNP (ref. 29), 1 053 SNP (ref. 31), and 1 918 and 2 818 SNP markers (ref. 32). Furthermore, a consensus map of apple was produced by merging a SNP genetic map with maps from earlier studies on eight FS families^34^ as follows: two based on SSR markers^24,25^ and six based on SSR and SNP markers.^36^ This consensus map consisted of 2 875 markers with a total genetic length of 1 991 cM. Finally, a multifamily single parent map was produced for the ‘Honeycrisp’ apple from three small progenies (318 individuals in total) having 1 091 SNPs (ref. 35). All of these genetic maps have a total size ranging from 1 282–1 991 cM. ^28,34^ The iGLMap is shorter than all these despite having the highest chance of false recombination due to data issues; and therefore an inflated genetic length due to being based on the largest number of markers, the largest number of FS families and the largest total number of individuals. This result once more proves the quality of the iGLMap and thereby the validity of our approach.

Further support for the quality of the iGLMap comes from the plots of the alternative plausible positions calculated by JoinMap. In the iGLMap, spots with uncertain marker order usually spanned less than 0.05 cM, and only occasionally spanned up to 0.1 cM. Such limited uncertainty was feasible thanks to the reduced level of missing values, the high level of data curation, and the high number of individuals that were included in our study, which theoretically allow a maximum resolution of ~0.02 cM when two HB markers segregate in all individuals.

The length of the iGLMap (1 267 cM) is shorter than both the direct and weighted average length of the 21 single family maps (1 305 cM), because additional data curation had been performed after the construction of the individual maps, during the data aggregation and the mapping of the obtained HB data, as described in the result section.

### Proposal for a re-orientation of the LG5 linkage map

In apple and pear, the convention for orienting linkage groups and pseudo-molecules is based on the genetic linkage map by Maliepaard *et al.*^2^ for ‘Prima’×‘Fiesta’. Maliepaard *et al.*^2^ were the first to report large stretches of homoeologous sequences as detected by multi-locus targeting RFLP clones. However, they did not use these first indications on homoeology for linkage group orientation. To date, those initial observations have been confirmed, and knowledge about duplication patterns have considerably increased.^36^ In apple, the orientation of the two chromosomes of the homoeologous pairs 5 and 10 and 13 and 16, where homoeology involves almost the entire chromosome, showed opposite orientation *sensu* Maliepaard *et al.*^2^ The orientation of LG13 has been modified to match that of LG16 by Liebhard *et al.*^67^ In the present work, we modified the orientation of the LG5 linkage map to match that of LG10 to facilitate future chromosome and genome comparison studies between homoeologous chromosomes within a species and synteny studies among species. We re-orientated LG5 and not LG10 because LG5 has been reported in less genetic studies on QTL and candidate genes identification in apple (Supplementary File S5). Due to the limited number of cases where putative QTLs and genes have been reported on LG5, we expect the benefits for future studies to offset the initial inconvenience generated by comparing our iGLMap with those of previous papers. In pear, the naming and orientation of linkage groups was based on apple^68^ having made use of the high synteny between these two crops.^68,69^ In the few QTL reports on pear, LG5 has been mentioned more frequently than LG10 (Supplementary File S5).

### The HB strategy, genotyping by sequencing and single families

The HB strategy contributed considerably to the quality of the IGLMap. This strategy is applicable to a wide range of genetic data and population types. Indeed, in this study, HB-sizing was essentially based on information from a draft genome sequence: Haploblock markers were created by the aggregation of segregation information from SNP that co-localized within a narrow physical window. The approach is also applicable with GBS derived data. In case of, Restriction site Associated DNA (RAD) sequencing,^70^ the various SNP from the same read may be used for the construction of a HB marker. The HB strategy may also be used on single families as it may considerably reduce computation time, especially in case of a large sized family and very high marker densities.

### Conclusion

The reliable marker order, high coverage and resolution of the iGLMap makes it a valid reference map for QTL mapping studies and the evaluation of genome assemblies. Therefore, the iGLMap can contribute to the enhancement of marker-assisted breeding approaches aimed at improving apple quality and productivity. The usefulness of the iGLMap has already been demonstrated in at least the following two occasions: i) its successful use in a recent pedigree-based analyses study identifying QTLs and possibly the underlying candidate genes for controlling chilling and heat requirements^18^ and ii) the improvement of the reference genome sequence of apple (https://www.rosaceae.org/species/malus/malus_x_domestica/genome_v3.0.a1).

Finally, the methodology presented here may be valuable for the construction of accurate high-density bi- and multi-parental integrated genetic linkage maps for any outbreeding species.

## Figures and Tables

**Table 1:** Identity and origin of the 21 full-sib families used for developing the integrated genetic linkage map (iGLMap)

*Family*	*Mother*	*Father*	*Number of seedlings*	*Sources*	*Previous studies*
12_B	‘Generos’	X-6417	48	INRA_Angers-France	^13,41,71,72^
12_E	‘Generos’	X-6683	58	INRA_Angers-France	^13,41,71,72^
12_F	X-3318	X-6564	48	INRA_Angers-France	^13,41,71,72^
12_I	X-3263	X-3259	47	INRA_Angers-France	^13,41,71,72^
12_J	X-3318	‘Galarina’	23	INRA_Angers-France	^13,41,71,72^
12_K	X-6679	X-6808	47	INRA_Angers-France	^13,41,71,72^
12_N	X-3305	X-3259	48	INRA_Angers-France	^13,41,71,72^
12_P	‘Rubinette’	X-3305	48	INRA_Angers-France	^13,41,71,72^
DiPr	‘Discovery’	‘Prima’	77	JKI-Germany	^13,33,41,71–74^
DLO.12	1980-15-25	1973-1-41	219	DLO-Netherlands	^27,75,76^
FuGa	‘Fuji’	‘Gala’	141	UNIBO-Italy	^41,71,77–79^
FuPi	‘Fuji’	‘Pinova’	91	RCL-Italy	^13,41,71,72^
GaPi	‘Gala’	‘Pinova’	40	RCL-Italy	^13,41,71,72^
I_BB	X-6417	X-6564	43	INRA_Angers-France	^13,41,71,72^
I_CC	X-6679	‘Dorianne’^a^	50	INRA_Angers-France	^13,41,71,72^
I_J	X-3318	X-3263	48	INRA_Angers-France	^13,41,71,72^
I_M	X-6683	X-6681	45	INRA_Angers-France	^13,41,71,72^
I_W	X-6398	X-6683	44	INRA_Angers-France	^13,18,41,71,72^
JoPr	‘Jonathan’	‘Prima’	174	DLO-Netherlands	^13,71,72^
PiRea	‘Pinova’	‘Reanda’	45	JKI-Germany	^13,41,71,72^
TeBr	‘Telamon’	‘Braeburn	202	KUL-Belgium	^80–83^
Total			1 586		

These overview data have been partially presented by Bianco *et al.*^22^ The number of genotyped seedlings has been updated after data curation in the current study during the construction of the iGLMap: 16 pairs of identical individuals were discovered across 6 families for which only 1 individual per pair was kept in the final data set; thus, a total of 16 identicals were removed. The involved families were 12_B (1 pair), DLO.12 (6 pairs), FuGa (1 pair), FuPi (1 pair), GaPi (3 pairs), I_M (1 pair), I_W (1 pair), JoPr (1 pair), and PiRea (1 pair). In addition, two individuals, 12_B058 and 12_J025 that showed a very high recombination rate (>5.0) in almost all linkage groups were considered out-crossers and excluded from the final data set. Most of these populations were part of the previous European project HiDRAS,^41^ and four of them derived from other previous studies as reported in the last column. Pedigrees of the X-numbered accessions are given in Bink *et al.*^13^

a X-6690.

**Table 2:** Summary of the 21 single-family genetic maps and the final integrated Genetic Map (iGLMap) by their genetic length (a) and number of markers (b)

*LG*	*12_B*	*12_E*	*12_F*	*12_I*	*12_J*	*12_K*	*12_N*	*12_P*	*DiPr*	*DLO.12*	*FuGa*	*FuPi*	*GaPi*	*I_BB*	*I_CC*	*I_J*	*I_M*	*I_W*	*JoPr*	*PiRe*	*TeBr*	*Pop Mean*	*iGLMap*
*(a)*																							
LG1	61	56	55	65	56	74	46	73	58	79	59	50	64	67	64	84	65	68	64	64	81	64±9.8	63.1
LG2	91	75	93	70	86	86	70	71	94	93	74	68	64	80	66	92	80	81	82	78	78	80±9.5	78.4
LG3	88	69	70	80	66	70	66	66	73	80	69	70	58	82	87	70	81	103	87	57	79	75±11.1	74
LG4	84	62	69	74	55	67	55	63	73	72	60	67	77	60	70	69	62	77	77	65	74	68±7.7	65.5
LG5	84	84	90	85	81	90	77	78	108	87	82	95	53	77	80	101	99	89	80	69	87	85±11.6	77.8
LG6	77	73	78	81	93	96	70	63	69	88	67	68	65	77	76	71	78	—	62	59	64	74±10.2	75.3
LG6P1^a^	—	—	—	—	—	—	—	—	—	—	—	—	—	—	—	—	—	32	—	—	—	—	—
LG6P2^a^	—	—	—	—	—	—	—	—	—	—	—	—	—	—	—	—	—	66	—	—	—	—	—
LG7	91	77	48	88	90	93	75	80	91	88	78	68	69	60	—	100	77	71	95	79	84	80±12.8	82.4
LG7P1^b^	—	—	—	—	—	—	—	—	—	—	—	—	—	—	60	—	—	—	—	—	—	—	—
LG8	70	78	55	68	68	63	53	58	77	98	65	56	66	65	65	68	63	66	84	72	67	68±10.2	68.5
LG9	64	68	72	76	76	95	68	68	90	68	66	57	61	76	72	62	89	72	73	56	67	71±10.1	67.1
LG10	90	82	93	78	95	105	80	77	90	94	77	76	57	86	74	100	78	102	76	72	87	84±11.7	81.3
LG11	80	79	77	68	89	155	79	83	91	96	72	89	67	64	85	85	88	100	83	62	85	85±19.1	80.9
LG12	92	61	90	59	75	82	76	62	69	77	63	61	54	69	64	77	57	82	68	56	62	69±11	65.4
LG13	85	57	62	77	111	121	70	57	70	78	63	68	78	54	81	79	71	61	66	54	65	73±17	71.4
LG14	62	54	67	61	62	62	70	67	73	79	57	55	49	53	70	67	87	74	78	52	64	65±9.9	64.4
LG15	145	103	131	114	143	128	111	103	108	140	112	111	105	135	110	116	101	124	115	106	122	118±13.8	112.2
LG16	68	72	85	74	87	71	79	53	61	69	70	79	68	74	64	74	82	47	69	84	76	72±10	67.5
LG16P1T^c^	—	—	—	—	—	—	—	—	—	—	—	—	—	—	—	—	—	33	—	—	—	—	—
LG17	92	66	79	75	102	96	61	77	66	74	64	55	71	76	—	70	73	86	80	67	76	75±11.8	71.8
LG17P1^a^	—	—	—	—	—	—	—	—	—	—	—	—	—	—	56	—	—	—	—	—	—	—	—
LG17P2^a^	—	—	—	—	—	—	—	—	—	—	—	—	—	42	—	—	—	—	—	—	—	—	
Total	1424	1216	1312	1290	1434	1551	1205	1197	1360	1460	1198	1195	1123	1256	1232	1384	1332	1418	1339	1153	1319	1305±113.3	1267
																							
*(b)*																							
LG1	305	276	296	178	287	189	206	225	340	289	275	314	340	254	146	270	276	241	407	270	265	269±59.5	672
LG2	453	320	519	468	553	407	537	543	523	431	507	528	548	431	410	385	436	410	597	491	548	478±70.5	1 042
LG3	413	420	444	489	362	426	382	460	368	523	418	465	452	480	427	268	323	424	509	474	495	430±62.8	951
LG4	291	309	354	353	190	308	320	447	368	338	422	356	346	357	292	413	392	372	453	405	436	358±62.4	804
LG5	390	519	491	464	357	512	527	578	548	500	511	544	483	592	408	364	442	485	639	534	594	499±75.6	1 114
LG6	230	261	362	241	354	255	263	243	378	255	382	345	385	352	293	303	359	—	408	409	433	326±65.6	741
LG6P1^a^	—	—	—	—	—	—	—	—	—	—	—	—	—	—	—	—	29	—	—	—	—	—	
LG6P2^a^	—	—	—	—	—	—	—	—	—	—	—	—	—	—	—	—	246	—	—	—	—	—	
LG7	279	288	252	230	293	288	187	208	268	217	334	304	226	340	—	224	374	295	357	234	365	278±55.7	674
LG7P1^b^	—	—	—	—	—	—	—	—	—	—	—	—	—	—	53	—	—	—	—	—	—	—	—
LG8	367	305	386	414	351	348	431	415	258	404	410	379	382	432	459	311	302	324	236	326	477	367±63.9	862
LG9	405	419	366	327	425	339	273	281	372	355	383	333	399	410	304	364	333	436	397	374	447	369±48.9	839
LG10	459	323	382	399	424	404	402	421	509	460	542	448	458	457	345	438	402	411	560	506	498	440±60.1	1 049
LG11	539	557	474	434	444	418	324	548	497	444	599	550	539	551	407	504	392	453	586	376	494	482±74.6	1 085
LG12	457	471	396	559	425	332	495	479	482	428	395	437	295	503	311	429	472	410	589	482	579	449±78.4	1 000
LG13	437	511	417	438	378	445	397	431	447	333	357	391	432	428	303	410	455	291	567	415	473	417±63.7	844
LG14	414	414	432	327	417	416	252	353	364	325	410	438	436	389	303	386	343	380	382	381	380	378±47.8	813
LG15	681	663	483	520	481	508	656	576	578	424	670	641	588	713	498	561	713	481	826	645	596	595±99.9	1 260
LG16	314	436	391	452	273	458	435	383	426	466	429	398	431	459	384	396	362	250	555	466	419	409±68.6	834
LG16P1T^c^	—	—	—	—	—	—	—	—	—	—	—	—	—	—	—	—	—	94	—	—	—	—	—
LG17	314	324	307	310	337	251	150	247	370	301	379	367	163	352	—	354	312	318	386	360	419	316±69.0	833
LG17P1^a^	—	—	—	—	—	—	—	—	—	—	—	—	—	—	55	—	—	—	—	—	—	—	—
LG17P2^a^	—	—	—	—	—	—	—	—	—	—	—	—	—	—	172	—	—	—	—	—	—	—	—
Total	6748	6816	6752	6603	6351^d^	6 304	6237	6838	7096	6493	7423	7238	6903	7500	5570	6380	6688	6350	8454	7148	7918	6848±634	15 417
Avg. dist. (cM)	0.21	0.18	0.19	0.2	0.23	0.25	0.19	0.18	0.19	0.22	0.16	0.17	0.16	0.17	0.22	0.22	0.2	0.22	0.16	0.16	0.17	0.19	

The size of the genetic maps in cM (a) and the number of SNP markers mapped (b), are reported for each of the 21 full-sib families, together with average values (±s.d.) across families (Pop Mean) and the final integrated Genetic Linkage Map (iGLMap). The table includes some single-parent maps that could not be integrated due to the lack of bi-parental markers (LG6 and LG16 in I_W, and LG7 and LG17 in I_CC). Average marker distance (Avg. dist.), is estimated as total genetic length divided by the total number of markers, is reported at the bottom of the table.

a The parental maps of LG6 and LG17, in family I_W and I_CC, respectively, could not be integrated due to the absence of bi-parental markers and had to be considered separately. Their total length is therefore an estimate, derived by the sum of the paternal maps (cM) to the non-overlapping region of the maternal map, obtained from the alignment of the single-parent maps to the iGLMap.

b In family I_CC, the genetic maps of LG7 is given by the maternal parent data only, as the paternal parent did not show any polymorphic marker.

c The top part of the LG16 maternal map in family I_W (LG16P1T) could not be integrated to the entire map due to homozygosity in the paternal LG16 for that region.

d For family 12_J, only 2,969 of the 6,351 markers were used in the construction of the iGLMap as a set of 3382 identical markers *sensu* JoinMap had been overlooked due to an administrative error. The identity of these markers is given in Supplementary File 2, together with their genetic positions on the bi-parental map and their progeny genotypes for family 12_J.

**Table 5:** Final integrated Genetic Linkage Map (iGLMap) and core map (values in brackets), summarized per linkage group (LG) by number of HB-markers, genetic length, average and maximum interval between adjacent markers

*iGLMap (core map)*					
*LG*	*Number HBs*	*Number individual SNPs*	*LG length in cM*	*Average interval in cM±s.d.*	*Maximum interval in cM*
LG1	132 (123)	43 (16)	63.08 (63.73)	0.36±0.37 (0.46±0.50)	1.68 (3.42)
LG2	181 (170)	63 (32)	78.42 (77.73)	0.32±0.27 (0.39±0.34)	1.42 (1.47)
LG3	183 (171)	39 (23)	73.95 (76.43)	0.31±0.22 (0.40±0.33)	1.53 (1.99)
LG4	139 (133)	30 (18)	65.51 (66.10)	0.39±0.37 (0.44±0.40)	1.84 (1.82)
LG5	201 (192)	40 (18)	77.84 (78.66)	0.32±0.32 (0.38±0.45)	1.84 (3.45)
LG6	146 (135)	34 (15)	75.26 (76.60)	0.42±0.46 (0.52±0.55)	3.29 (2.99)
LG7	133 (121)	27 (12)	82.39 (82.83)	0.52±0.52 (0.63±0.70)	2.99 (5.20)
LG8	147 (135)	32 (15)	68.52 (66.04)	0.38±0.42 (0.44±0.45)	2.6 (2.55)
LG9	151 (144)	50 (28)	67.08 (66.79)	0.34±0.34 (0.39±0.39)	2.65 (2.58)
LG10	186 (174)	45 (23)	81.3 (81.93)	0.35±0.39 (0.42±0.46)	2.66 (2.71)
LG11	186 (176)	45 (20)	80.94 (83.22)	0.35±0.34 (0.43±0.40)	1.96 (2.27)
LG12	169 (161)	39 (26)	65.44 (66.26)	0.32±0.28 (0.36±0.32)	1.53 (1.50)
LG13	164 (149)	29 (17)	71.36 (71.50)	0.37±0.36 (0.43±0.44)	2.59 (2.65)
LG14	145 (135)	34 (15)	64.39 (66.03)	0.36±0.41 (0.44±0.45)	3.02 (3.13)
LG15	234 (227)	69 (34)	112.15 (112.98)	0.37±0.40 (0.44±0.52)	3.17 (3.30)
LG16	151 (150)	30 (21)	67.48 (68.43)	0.38±0.45 (0.40±0.53)	2.92 (3.90)
LG17	149 (135)	27 (11)	71.80 (73.68)	0.41±0.41 (0.51±0.58)	2.31 (4.10)
Total	2797 (2631)	676 (344)	1266.88 (1278.84)		
Average	165 (155)	40 (20)	74.52 (75.23)	0.37 (0.44)	

**Table 4:** Summary of designed and mapped HaploBlocks (HBs)

*SNPs source*^a^	*Size (kbp)*	*No. designed*	*No. mapped*	*Max no. of clustered SNPs*
FB	10	1696	1670	11
FB+IRSC	20	642	640	15
IRSC	100	499	487	8
Total		2837	2797	

Abbreviations: FB, FruitBreedomics; HB, HaploBlock; IRSC, International Rosaceae SNP Consortium; SNP, single-nucleotide polymorphism.

The number of designed and mapped HBs is reported for each SNP source type together with the physical distance range that defined the HB. The range of clustered SNPs per HB went from two up to 15 SNPs (FB+IRSC markers).

a See legend of Table 3.

**Table 3:** Summary of the number of available, validated and mapped SNPs markers

*Source*	*Available on the 20 K array*	*Validated SNPs*	*iGL mapped SNPs*
FB	14 628	12 508	12 349
Customized markers	103	60	53
IRSC–RosBREED	2750	2601	2481
IRSC–GDsnp	538	528	520
Multi-locus SNPs replicates		115	14
Total	18 019	15 812	15 417

Abbreviations: FB, FruitBreedomics, designed in the 20 K Infinium SNP array development;^22^ IRSC, RosBREED and GDsnp markers, from the previously developed International Rosaceae SNP Consortium (IRSC) 8 K Infinium SNP array by Chagnè *et al.*;^37^ SNP, single-nucleotide polymorphism.

Starting from the available markers as present on the 20 K Infinium SNP array,^22^ the number of validated SNPs (as the number of markers that mapped in at least one of the 21 individual families) and the number of SNP markers successful mapped on the integrated Genetic Linkage Map (iGL-mapped SNPs) are reported for each source type. Due to the presence of 111 multi-locus SNPs, the total count of validated and mapped SNPs includes multi-locus SNPs replicates as shown in the table.
