# Supplementary material for: A high-density, multi-parental SNP genetic map on apple validates a new mapping approach for outcrossing species
Source: Hortic Res. 2016 Nov 23;3:16057–. doi: 10.1038/hortres.2016.57 (PMC5120355; doi:10.1038/hortres.2016.57)
Supplement: Supplementary Information [file hortres201657-s1.doc]

**Content file for Supplementary Information**

**README file** for Di Pierro et al. “A high-density, multi-parental SNP genetic map on apple validates a new mapping approach for outcrossing species”. (.docx)

**Table S1 –** lists the SNP and HaploBlock markers providing their ID numbers and genetic location. (.xlsx)

**Table S2 –** lists the multi-locus SNPs and reports the linkage groups on which they map. (.xlsx)

**Figure S1 -** present projections on the integrated Genetic Linkage Map of the parental maps of I_W family LG6, I_W family LG16, and I_CC family LG17, respectively. (.pdf)

**Figure S2 -** presents the Haploblock distribution per source type and number of SNPs. (.pdf)

**Figure S3 -** presents the final consensus integrated Genetic Linkage Map (iGLMap). (.pdf)

**File S1 –** is a .zip folder that contains the Haplotype Aggregator input .csv files for the 21 full-sib families providing individual genotypes. (.pdf)

**File S2 –** contains the bi-parental genetic maps of each of the 21 full-sib families. (.xlsx)

**File S3 –** contains graphical genotyping visualizations for the final integrated BackCross-type dataset for each linkage group (from LG1 to LG17) of the iGLMap produced. (.xlsx)

**File S4 -** contains the *Plausible Positions* data that were generated by Join Map® for each linkage group of the iGLMap produced, and it also visualizes these data graphically. (.xlsx)

**File S5 –** gives an overview on genetic studies that report QTL or candidate genes on LG 5 and 10 in apple and pear. (.doc)
