# Supplementary figures and images for: A high-density, multi-parental SNP genetic map on apple validates a new mapping approach for outcrossing species
Source: Hortic Res. 2016 Nov 23;3:16057–. doi: 10.1038/hortres.2016.57 (PMC5120355; doi:10.1038/hortres.2016.57)

## Slide 1
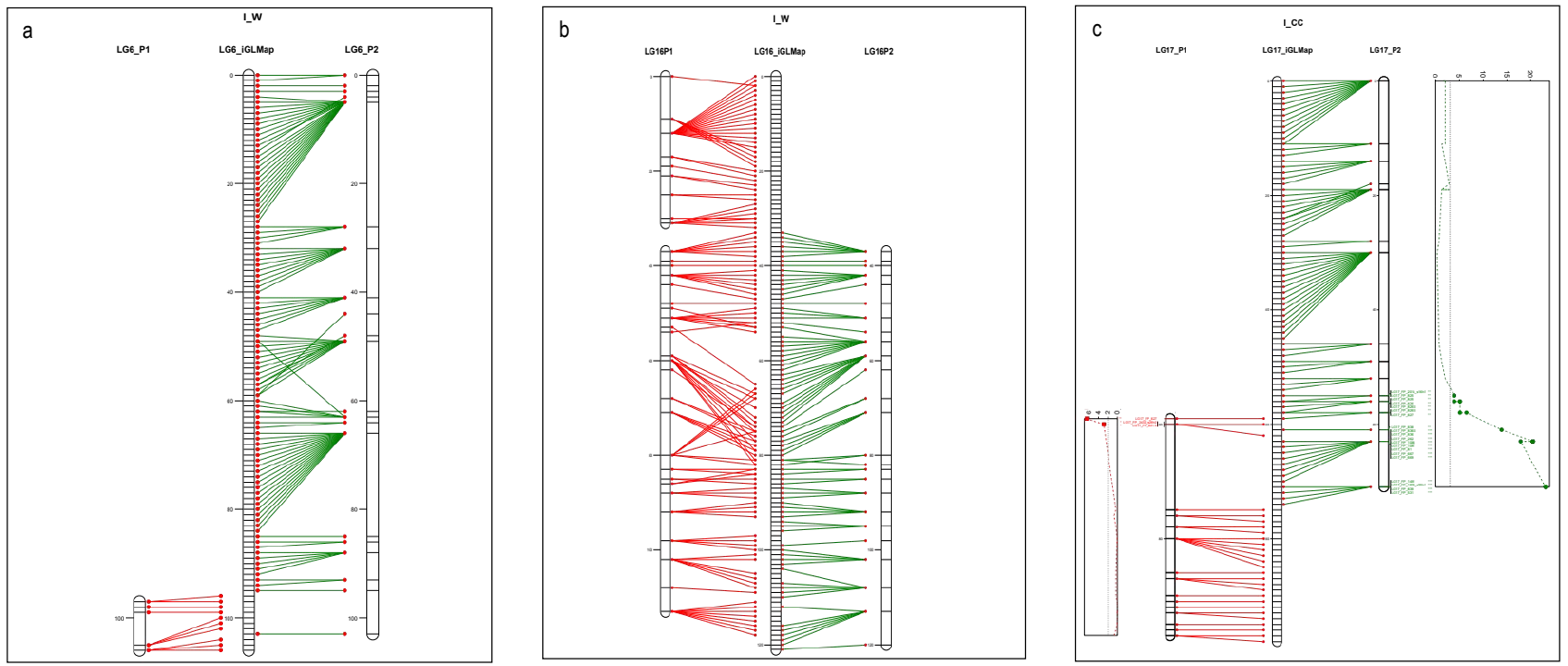

Supplement: Supplementary Figure S1 [file hortres201657-s2.ppt]

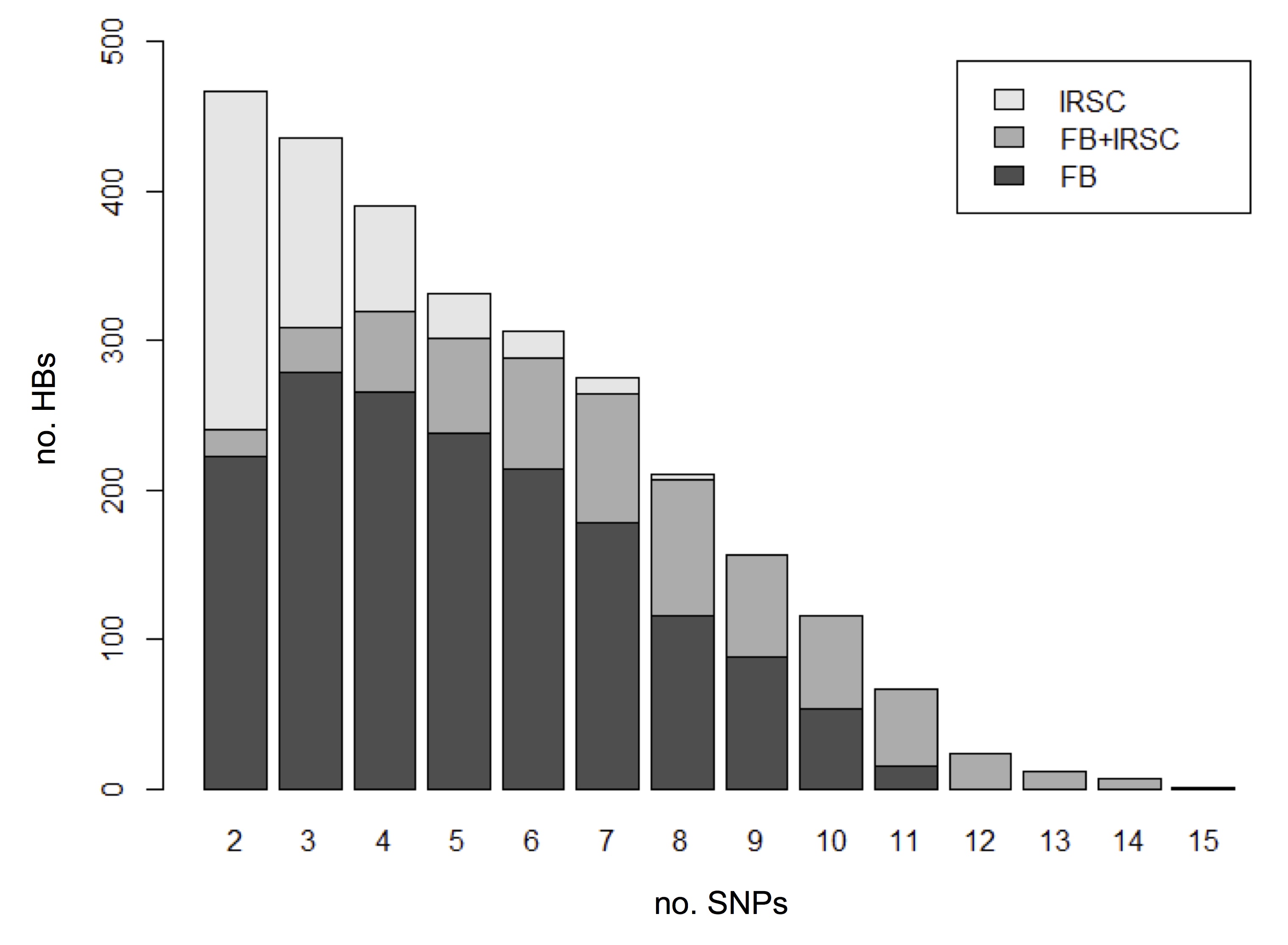

Supplement: Supplementary Figure S2 [file hortres201657-s3.jpg]
