# Supplementary material for: A high-density, multi-parental SNP genetic map on apple validates a new mapping approach for outcrossing species
Source: Hortic Res. 2016 Nov 23;3:16057–. doi: 10.1038/hortres.2016.57 (PMC5120355; doi:10.1038/hortres.2016.57)

## Slide 1
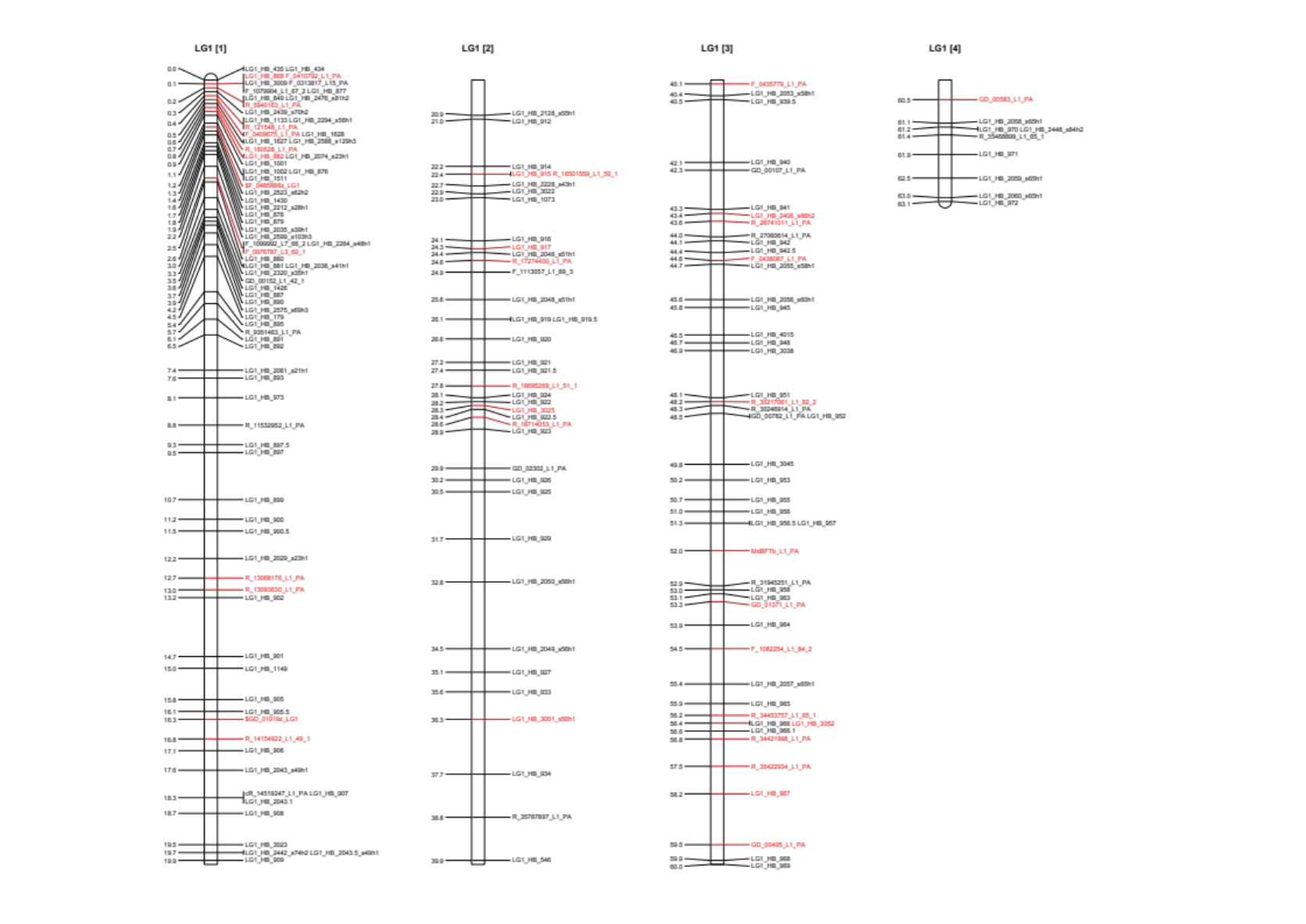

## Slide 2
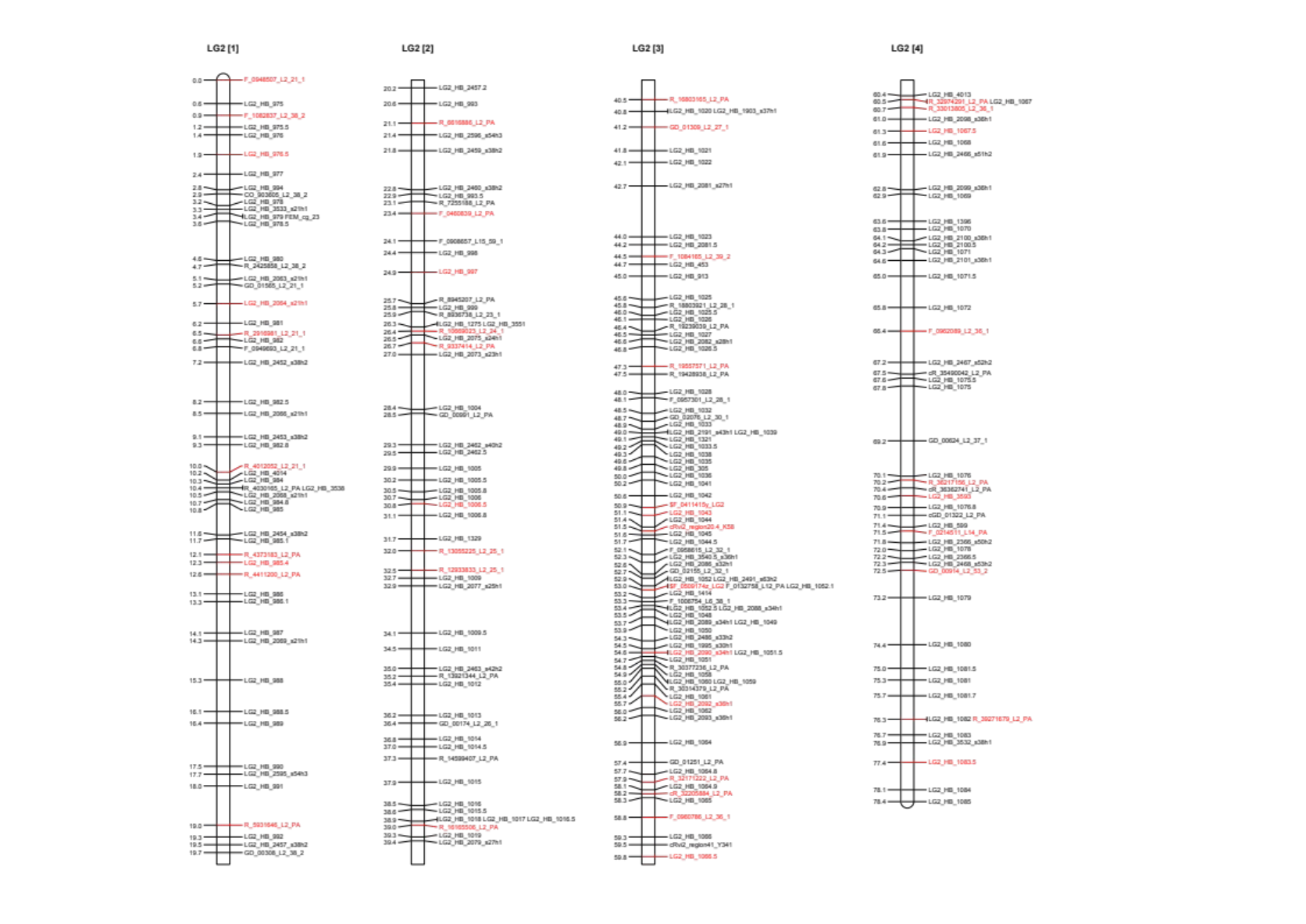

## Slide 3
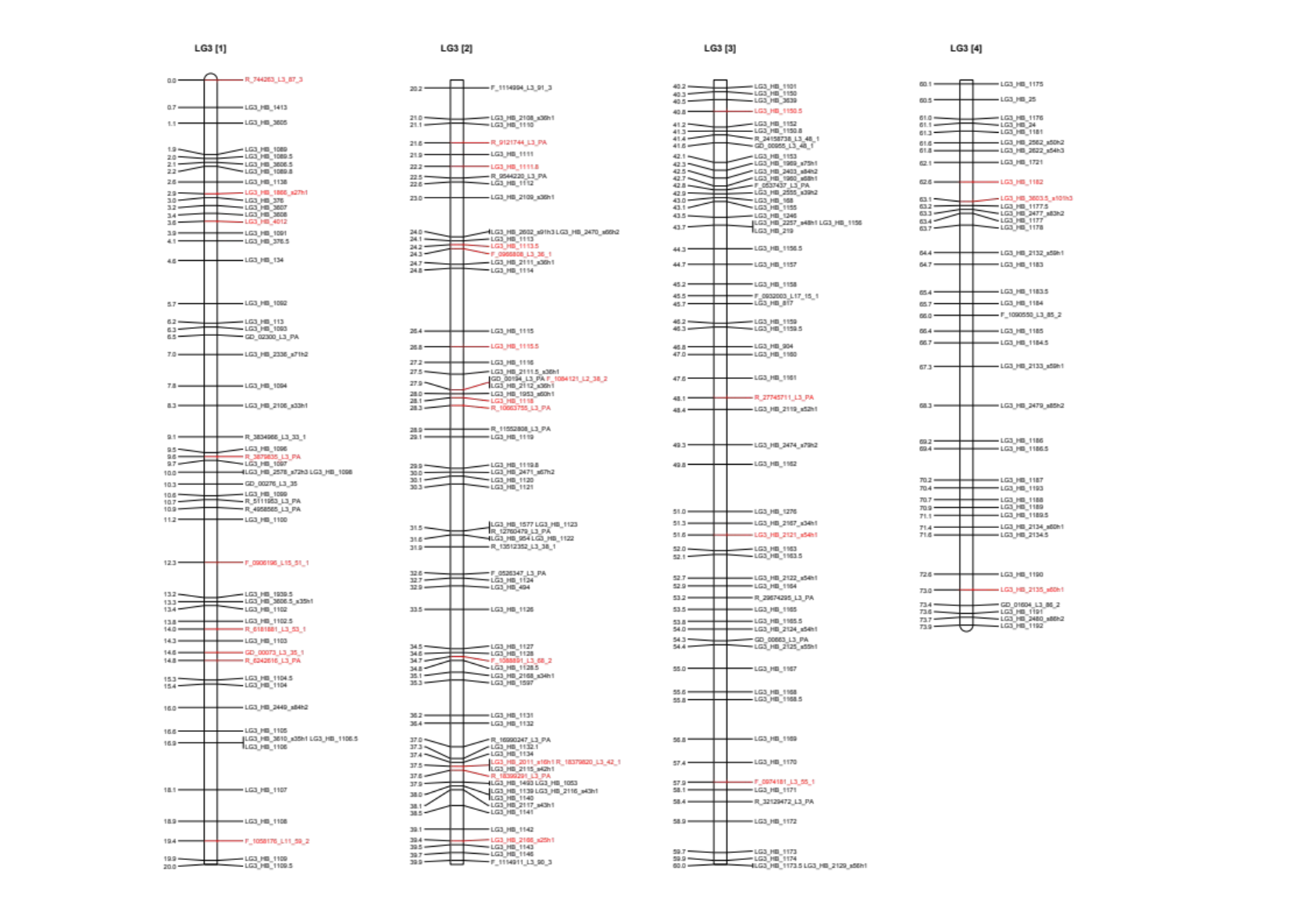

## Slide 4
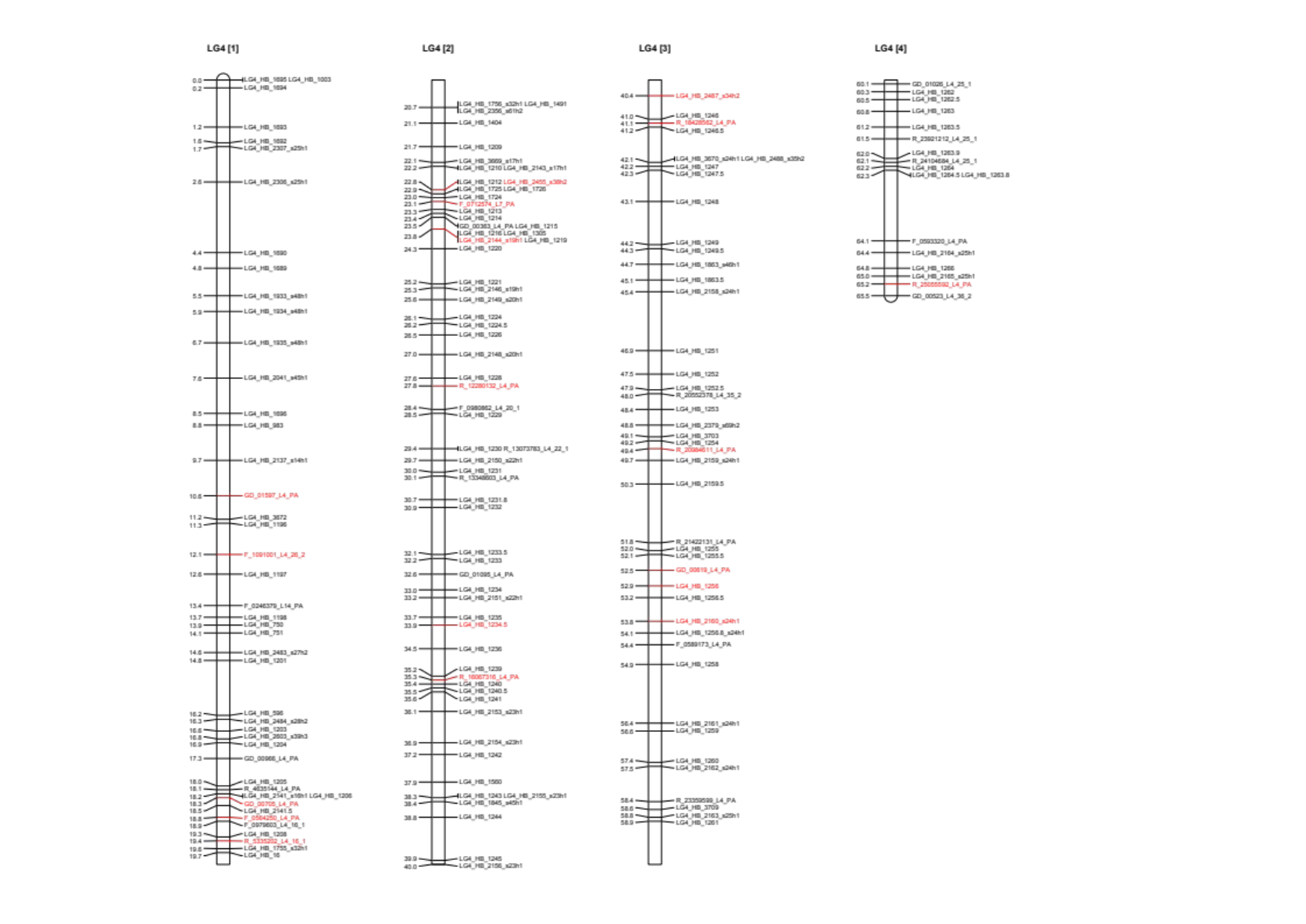

## Slide 5
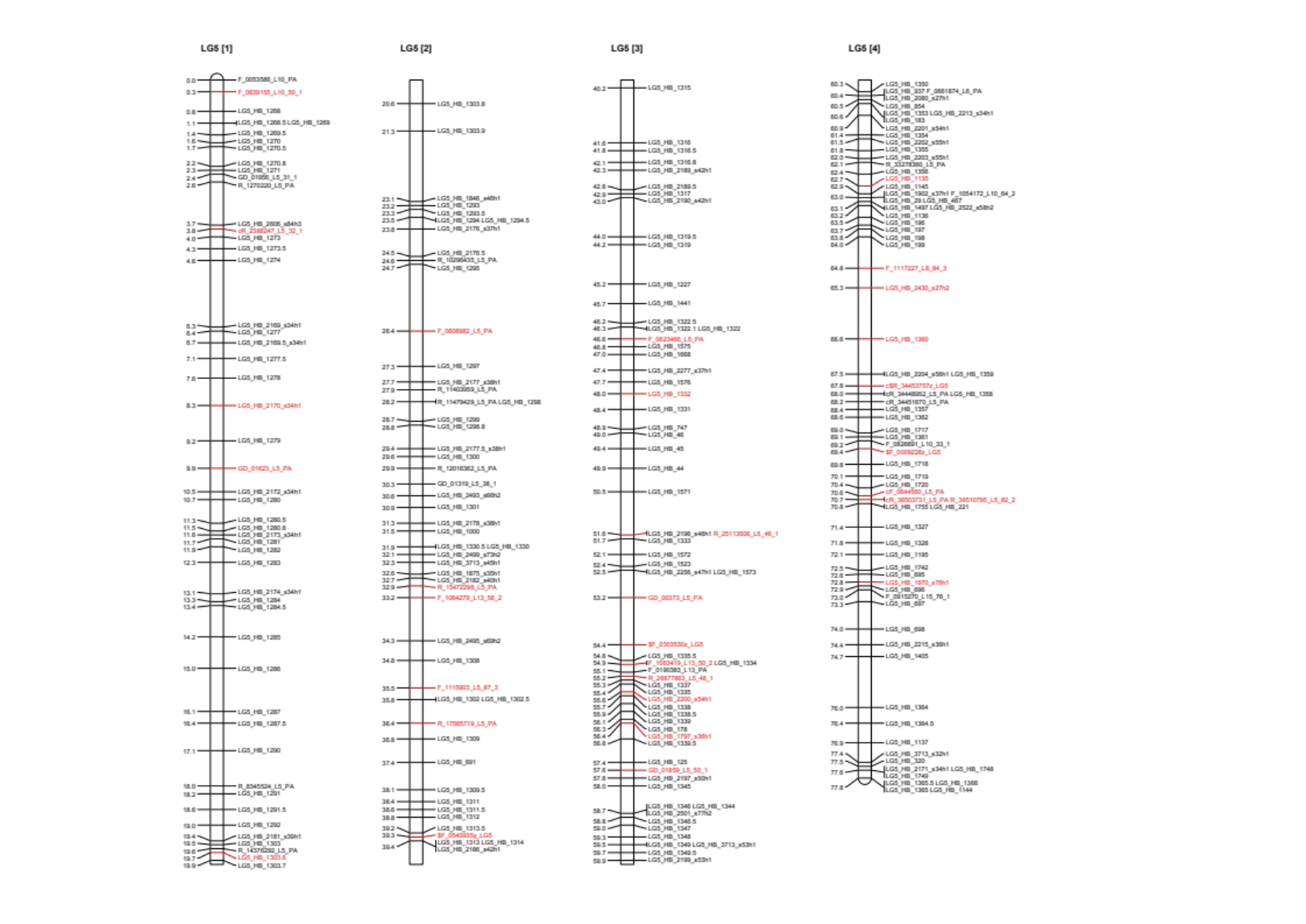

## Slide 6
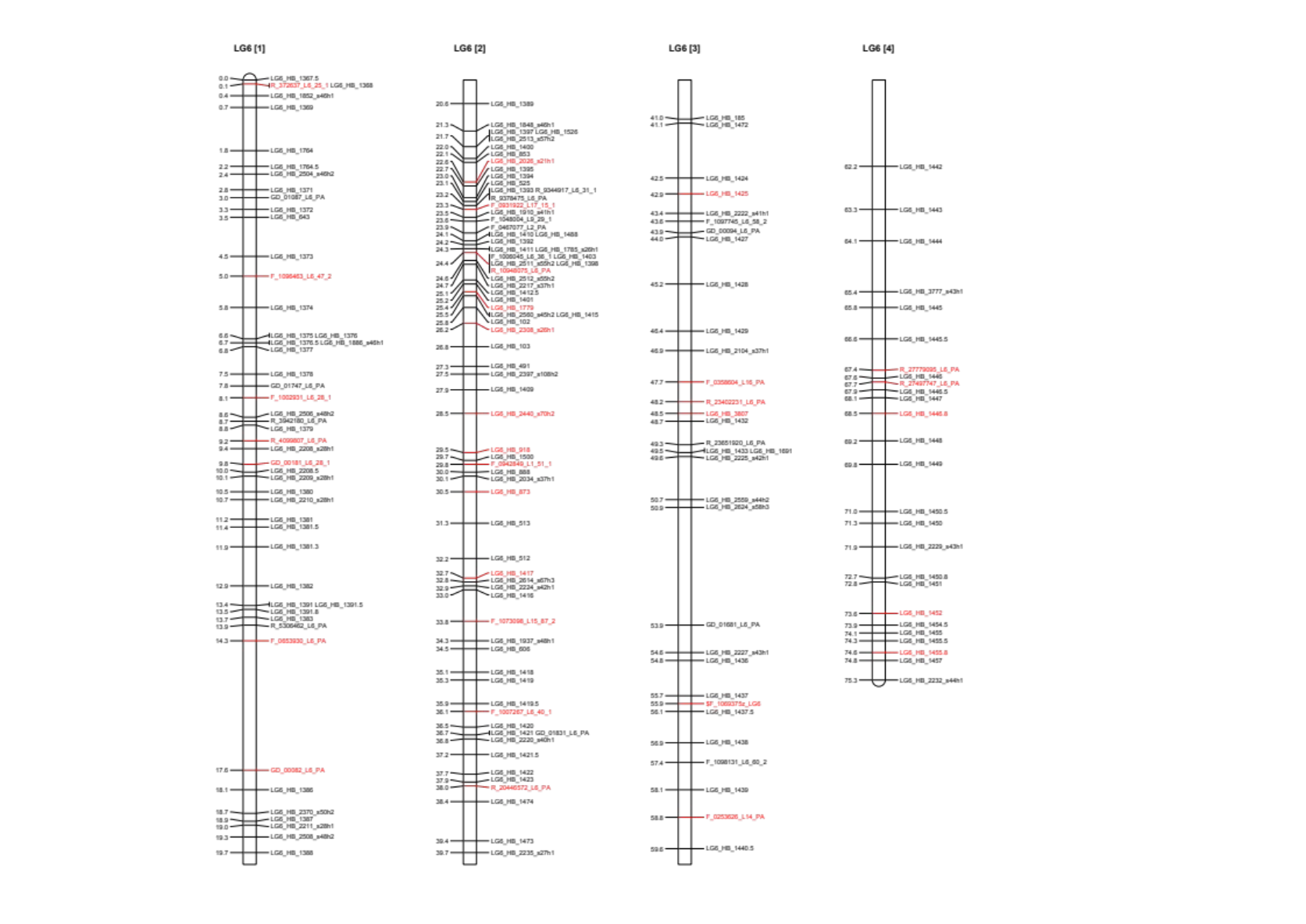

## Slide 7
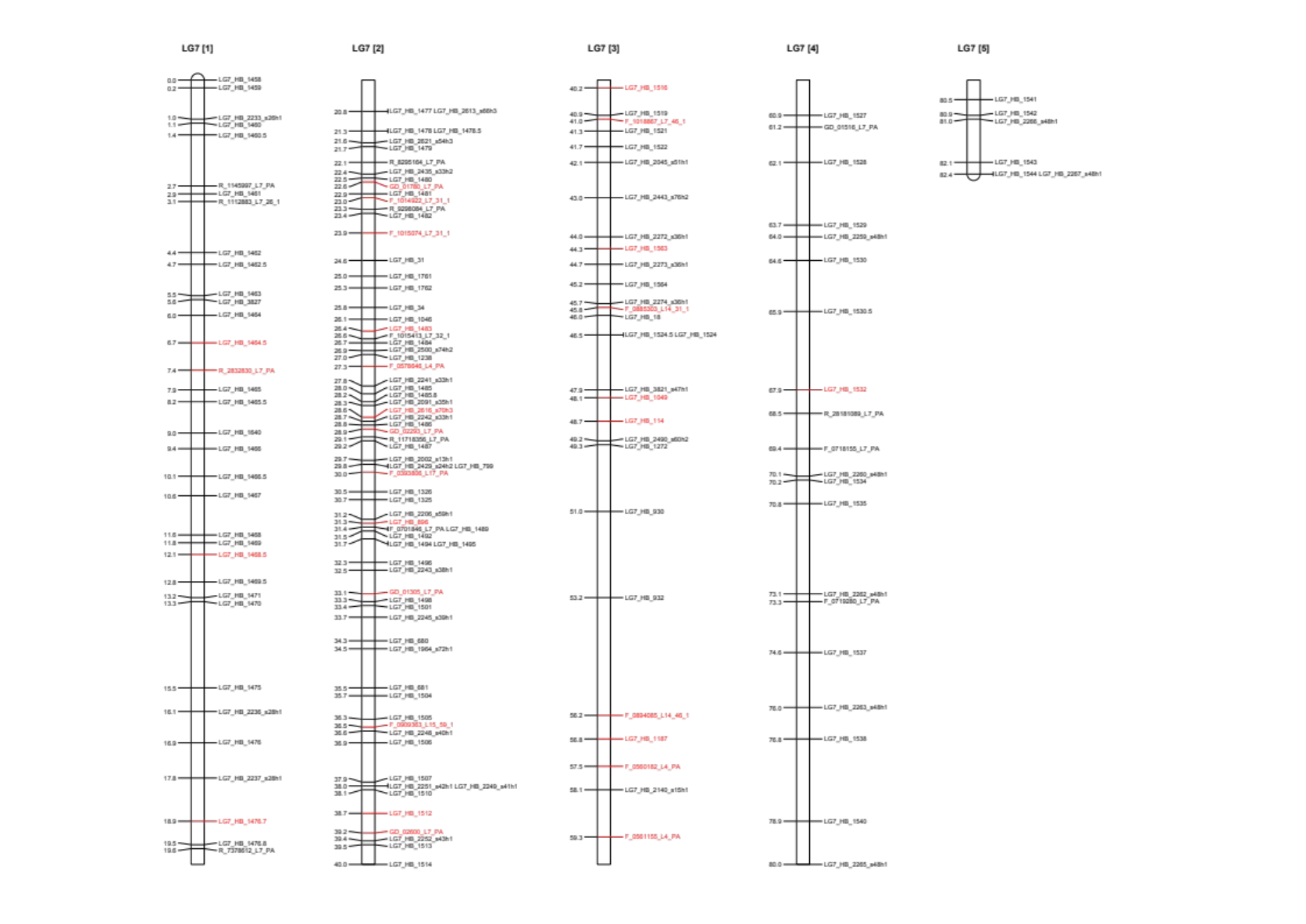

## Slide 8
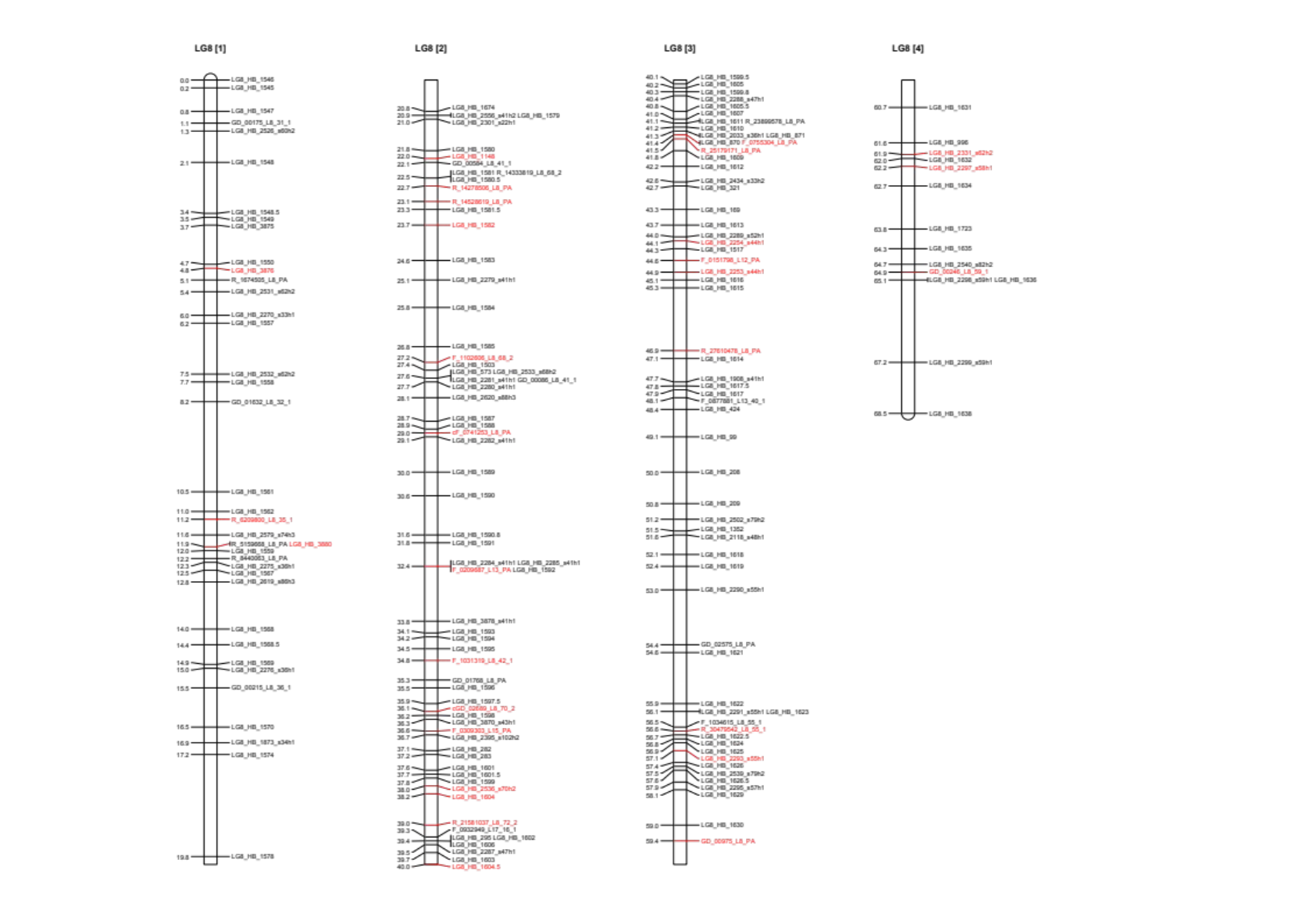

## Slide 9
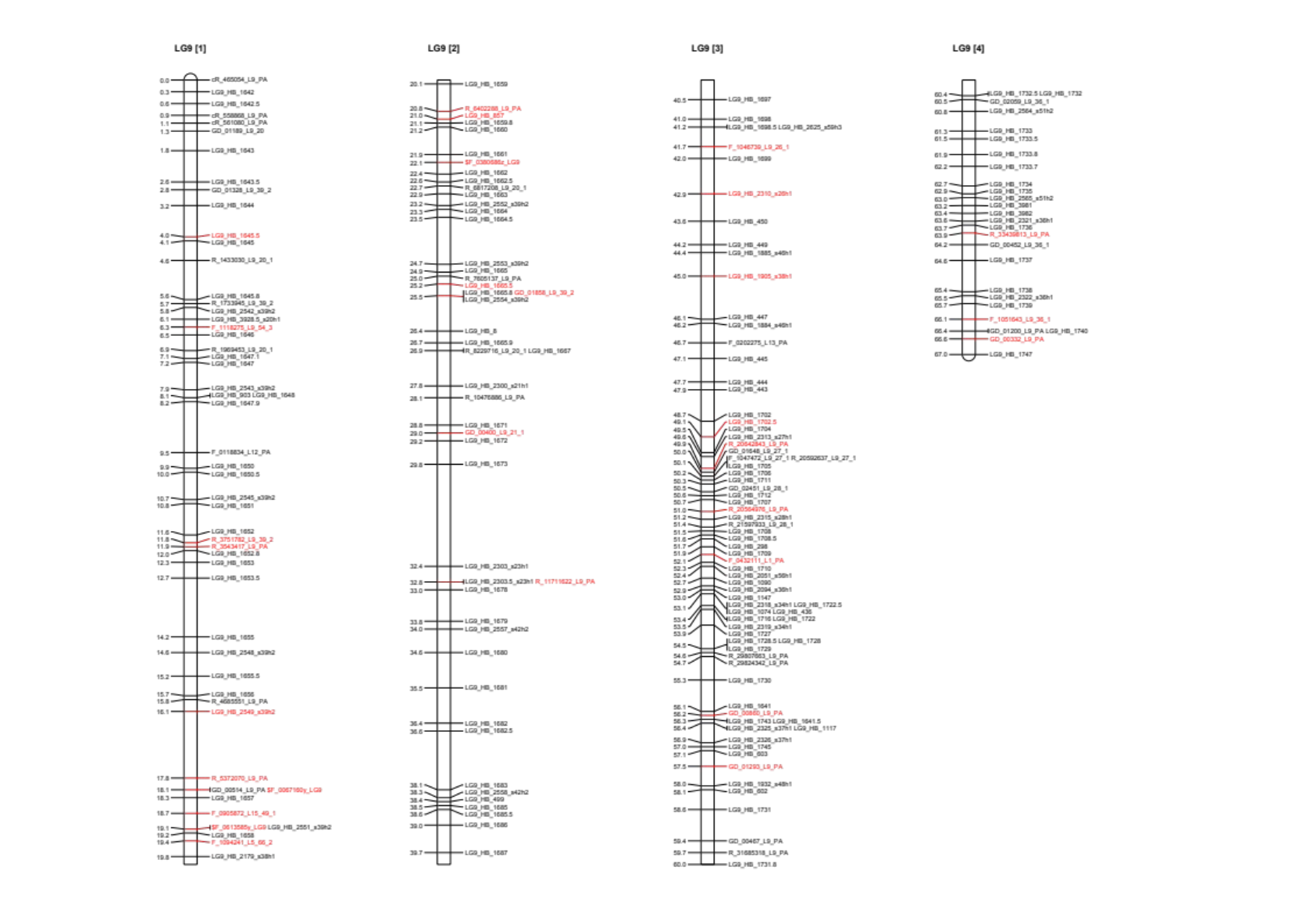

## Slide 10
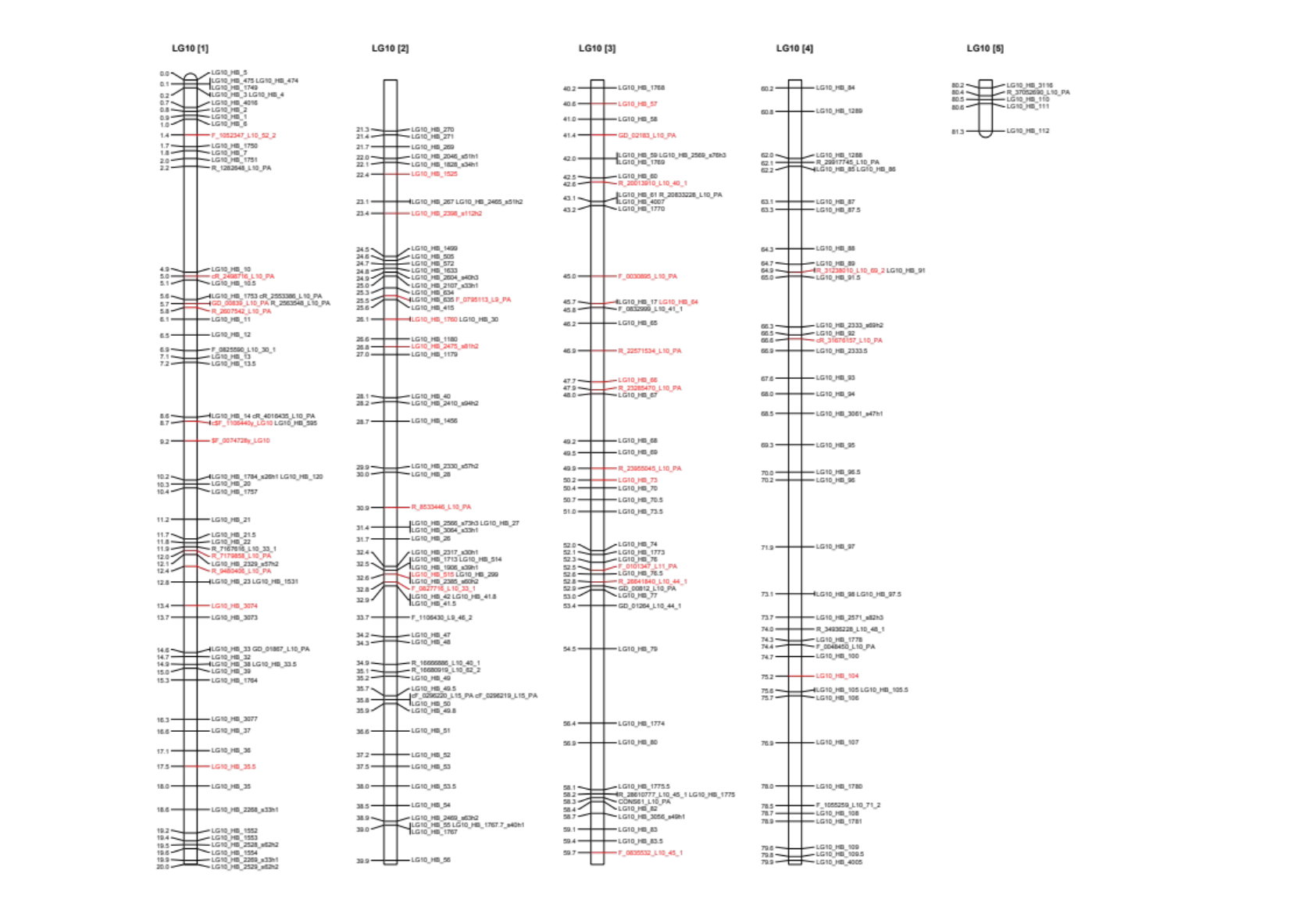

## Slide 11
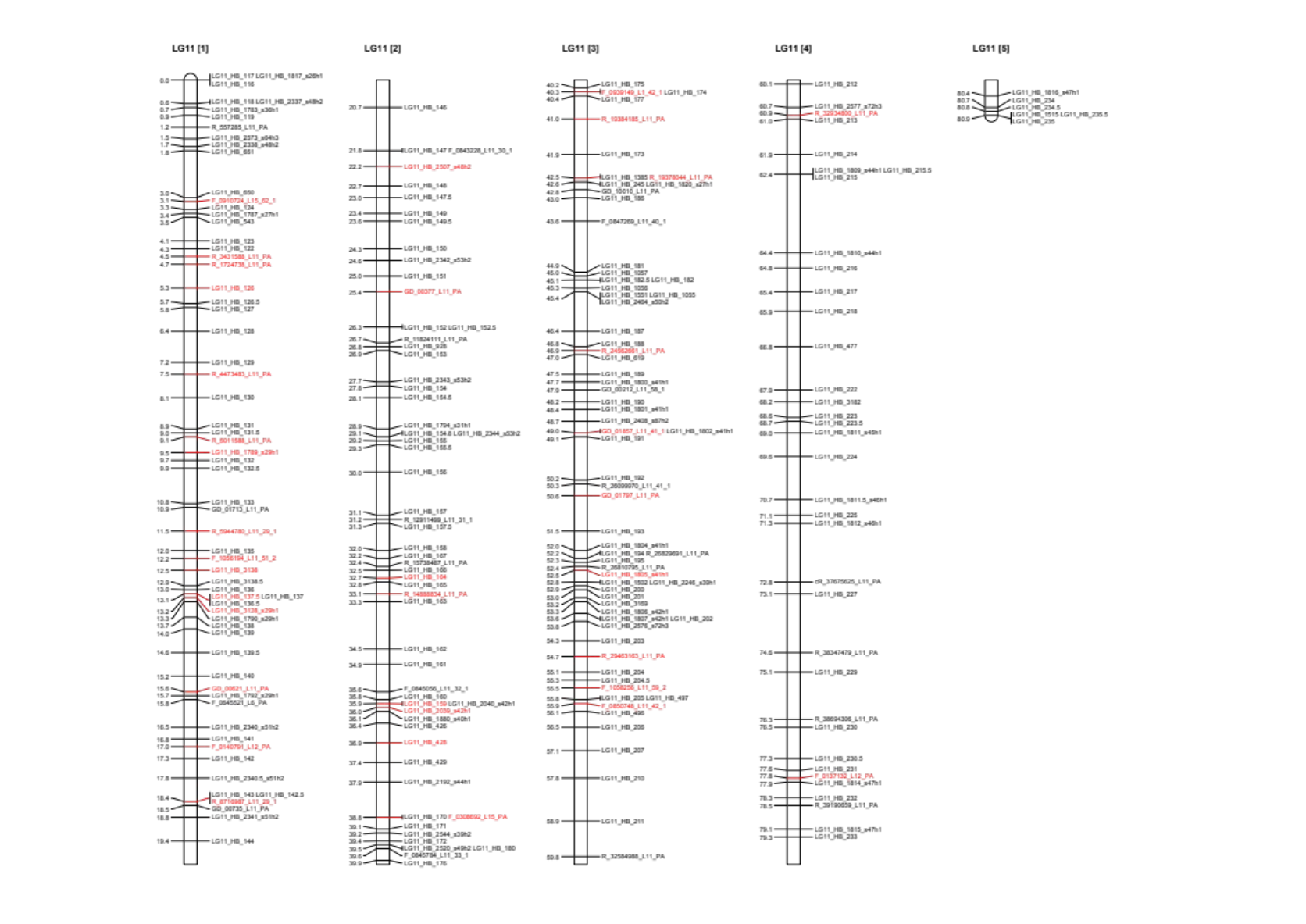

## Slide 12
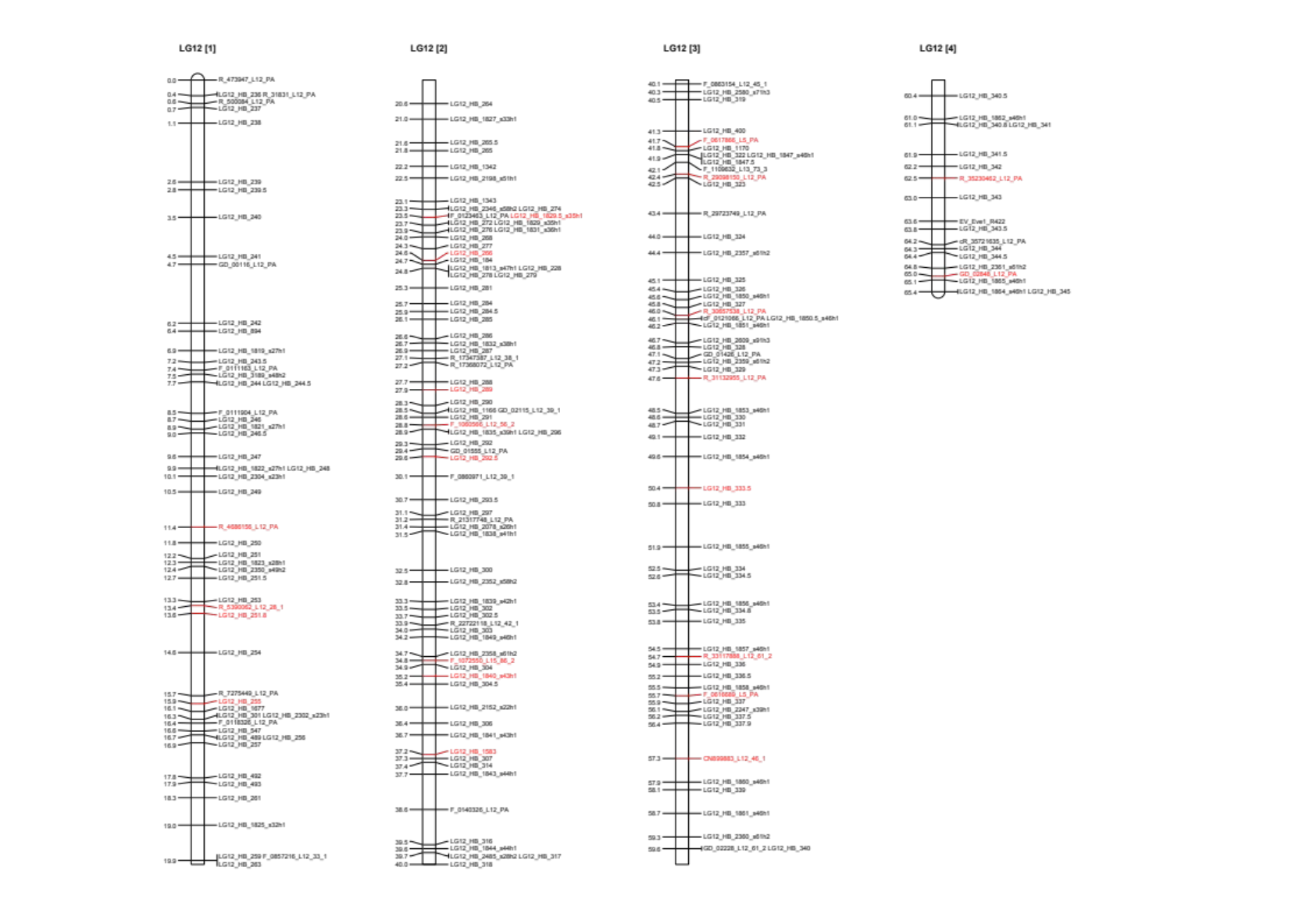

## Slide 13
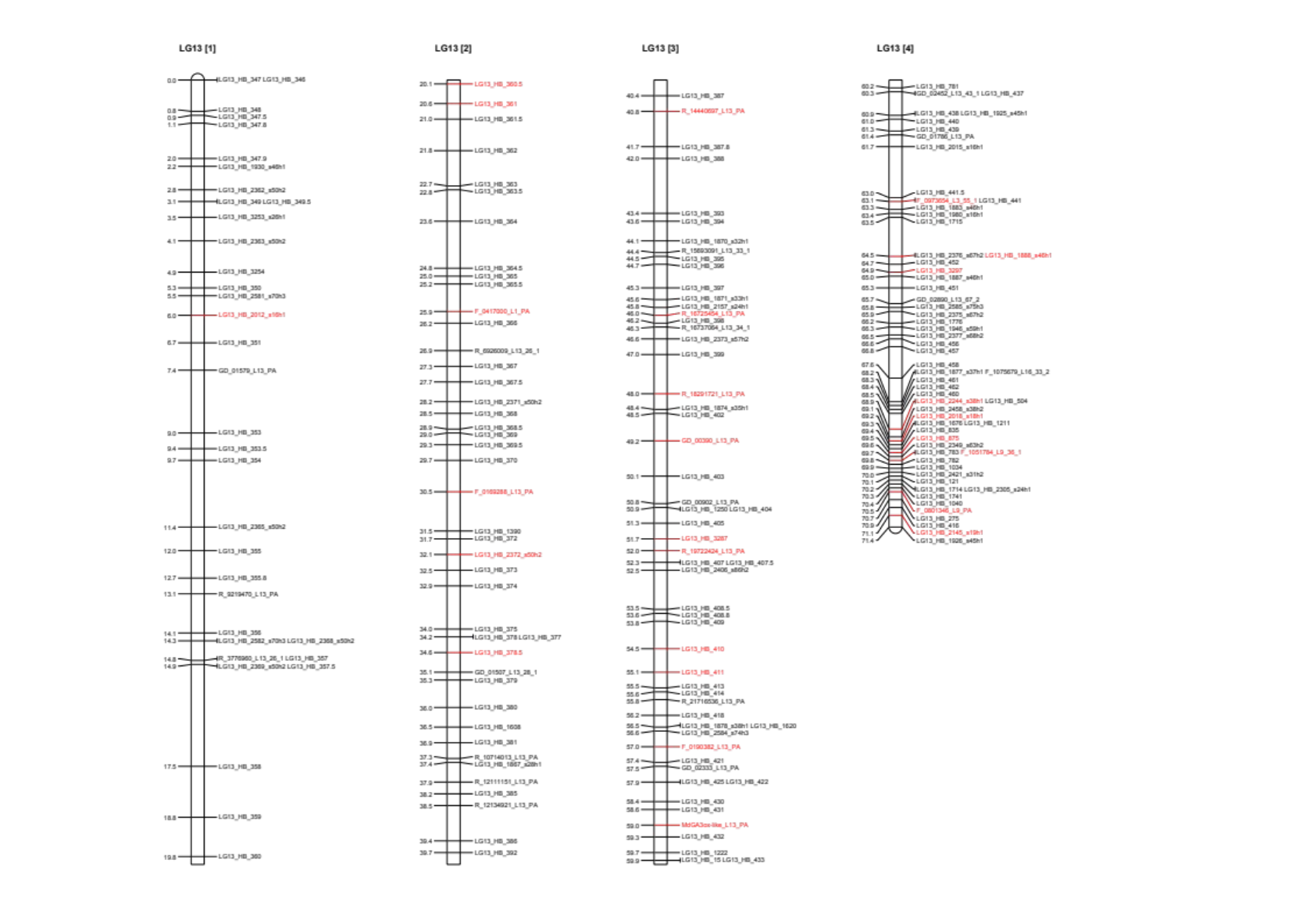

## Slide 14
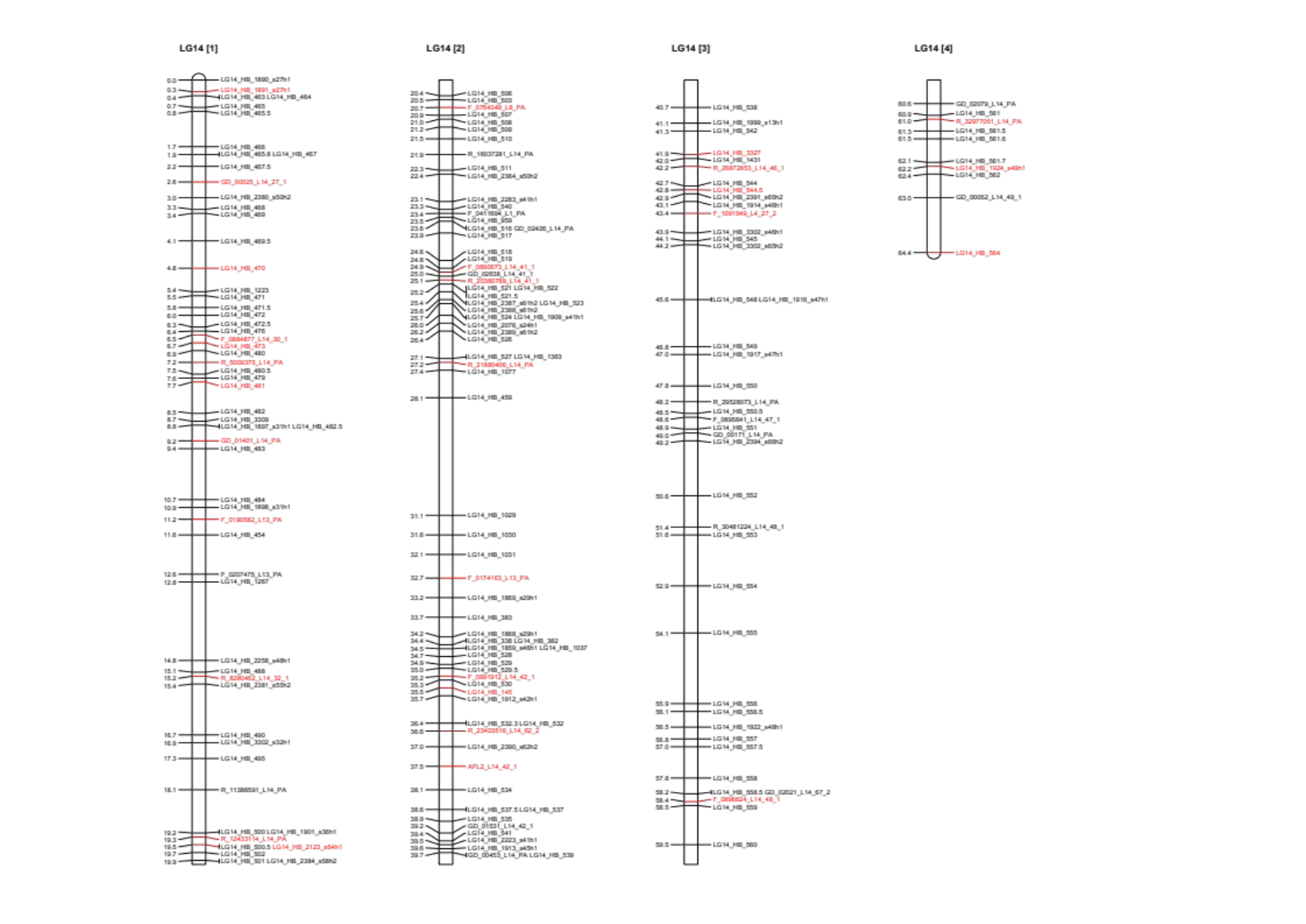

## Slide 15
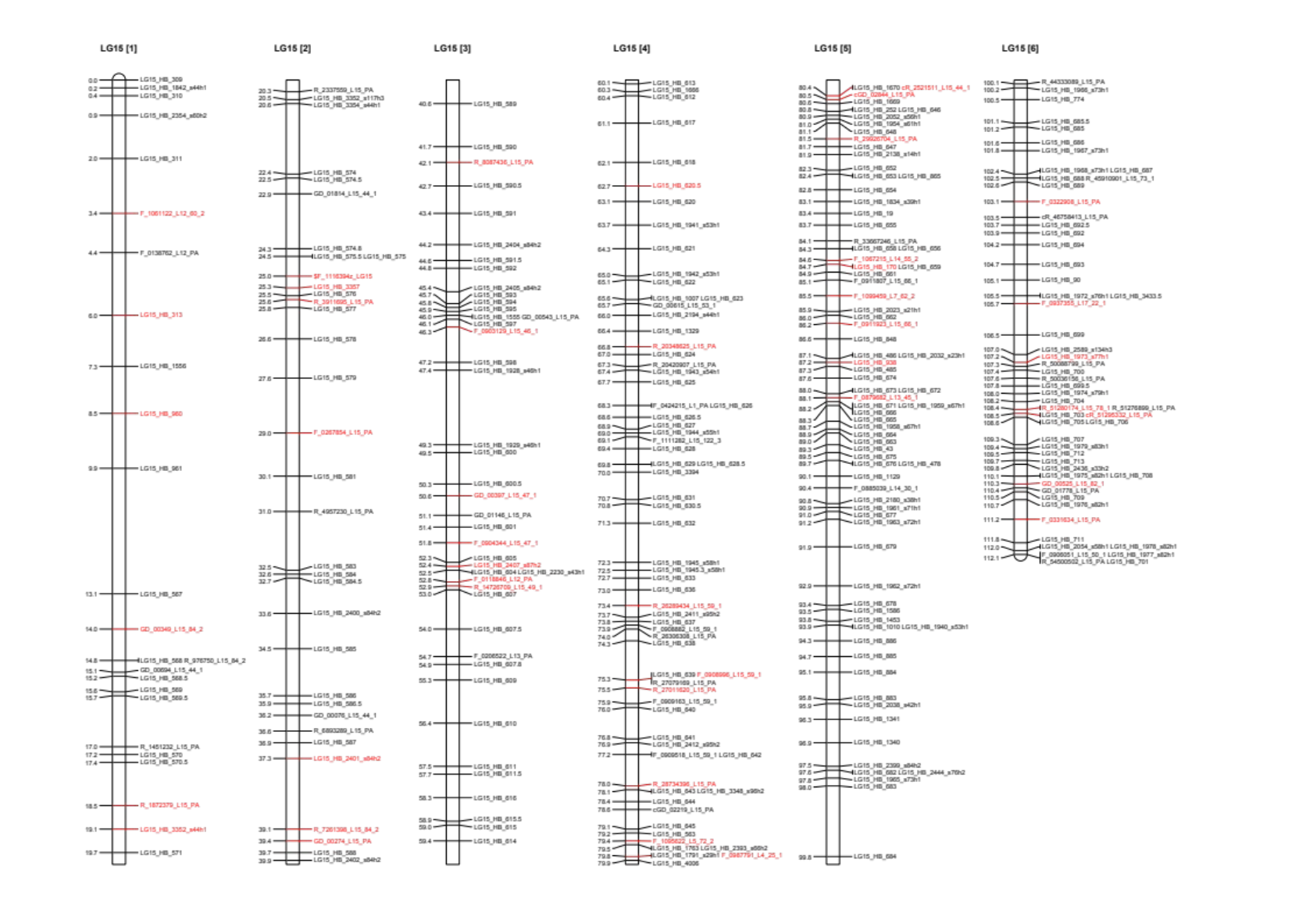

## Slide 16
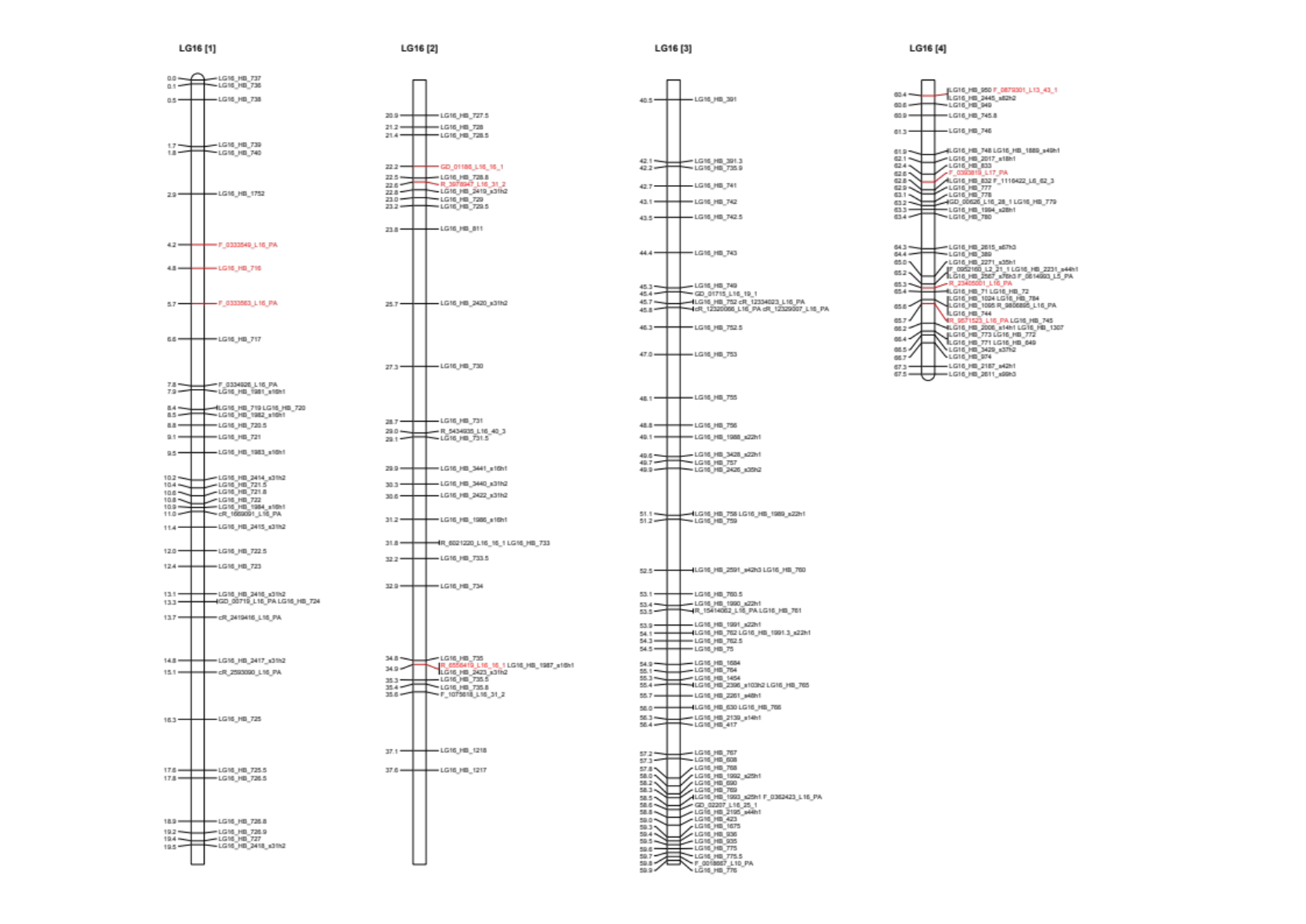

## Slide 17
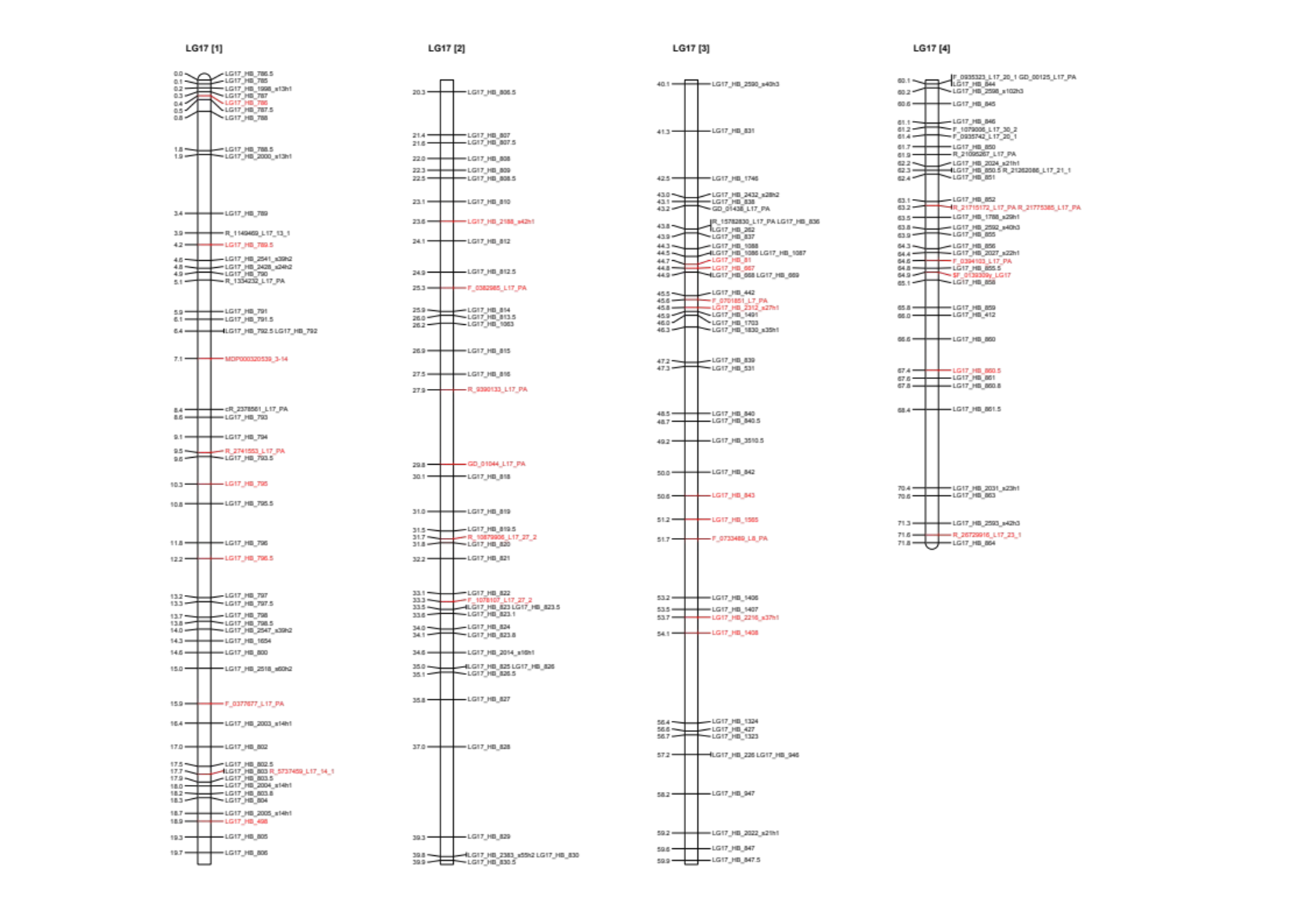

Supplement: Supplementary Figure S3 [file hortres201657-s4.ppt]
