# Supplementary material for: A high-density, multi-parental SNP genetic map on apple validates a new mapping approach for outcrossing species
Source: Hortic Res. 2016 Nov 23;3:16057–. doi: 10.1038/hortres.2016.57 (PMC5120355; doi:10.1038/hortres.2016.57)
Supplement: Supplementary File 5 [file hortres201657-s6.doc]

# SUPPLEMENTARY FILE 5

**Overview on genetic studies that report QTL or candidate genes on linkage group (LG) 5 and 10 in apple and pear.**

| **Crop** | **LG** | **Authors** | **Trait** | | |
| --- | --- | --- | --- | --- | --- |
|  |  |  | **Fruit quality** | **Disease Resistance** | **Other** |
| **Apple** | **5** | Liebhard et al. 2003 |  |  | Flowering time |
|  | **5** | Pilcher *et al.* 2008 |  |  | Dwarfing |
|  |  | Peil *et al.* 2011 |  | *Erwinia amylovora* |  |
|  |  | Devoghalaere *et al.* 2012 | Fruit weight |  |  |
|  | **10** | Hemmat *et al.* 1997 |  |  | Columnar grow |
|  |  | Conner *et al.* 1998 |  |  | Columnar grow |
|  |  | Maliepaard *et al*. 1998 | Firmness |  | Columnar grow |
|  |  | Maliepaard *et al*. 2001 | Firmness |  |  |
|  |  | Liebhard *et al*. 2003 |  |  | Height increment bud-grafts |
|  |  | Tartarini *et al.* 2004 |  | *Venturia inaequalis* |  |
|  |  | Costa *et al*. 2005 | Firmness |  |  |
|  |  | Davey *et al*. 2006 | Vitamin content |  |  |
|  |  | Calenge & Durel 2006 |  | *Podosphaera leucotricha* |  |
|  |  | Zhu and Barritt 2008 | Firmness |  |  |
|  |  | Costa *et al*. 2010 | Firmness |  |  |
|  |  | Kumar *et al*. 2012 | Firmness |  |  |
|  |  | Longhi *et al.* 2012 | Firmness |  |  |
|  |  | Longhi *et al.* 2013a | Firmness |  |  |
|  |  | Longhi *et al.* 2013b | Firmness |  |  |
|  |  | Wolters *et al*. 2013 |  |  | Columnar grow |
|  |  | Bink *et al*. 2014 | Firmness |  |  |
|  |  | Emeriewen *et al.* 2015 |  | *Erwinia amylovora* |  |
|  |  | Allard *et al*. 2016 |  |  | Bud break & Flowering time |
|  | **5&10** |  |  |  |  |
|  | 5 | King et al. 2000 | Hardness & Breakdown of fruit |  |  |
|  | 10 | King et al. 2000 | Firmness, Crispness, Breakdown of fruit |  |  |
|  | 10 | King *et al*. 2001 | Firmness |  |  |
|  | 5 | Kenis *et al*. 2008 | Diameter |  |  |
|  | 10 | Kenis *et al*. 2008 | Diameter, Weight, Firmness Brix, Acidity |  |  |
|  | 5 | Kumar *et al*. 2015 | Volatiles |  |  |
|  | 10 | Kumar *et al*. 2015 | Volatiles |  |  |
|  | 5 | Foster *et al. 2015* |  |  | Dwarfing |
|  | 10 | Foster *et al. 2015* |  |  | Dwarfing |
| **Pear** | **5** | Won *et al. 2014* |  | *Venturia pirina* |  |
|  |  | Knäbel *et al.* 2015 |  |  | Dwarfing & precocity |
|  |  | Montanari *et al*. 2015 |  | *Cacopsylla pyri* |  |
|  |  | Montanari *et al*. 2016 |  |  | Postzygotic hybrid necrosis |
|  | **10** | Won *et al. 2014* |  | *Venturia pirina* |  |
|  | **5&10** | - |  |  |  |

REFERENCES APPLE

Allard A, Bink MCAM, Martinez S, *et al*. Detecting QTLs and putative candidate genes involved in budbreak and flowering time in an apple multiparental population. *. Exp Bot* 2016; erw130.

Bink MCAM, Jansen J, Madduri M, *et al.* Bayesian QTL analyses using pedigreed families of an outcrossing species, with application to fruit firmness in apple. *Theor Appl Genet* 2014 ;127: 1073–1090.

Calenge F, Durel, C-E. Both stable and unstable QTLs for resistance to powdery mildew are detected in apple after four years of field assessments. *Mol Breed* 2006;17: 1-11; doi: 10.1007/s11032-006-9004-7.

Conner PJ, Brown SK, Weeden NF, *et al.* Molecular marker analysis of quantitative traits for growth and development in juvenile apple trees. *Theor Appl Genet* 1998; 96: 1027–1035.

Costa F, Peace CP, Stella S, *et al*. QTL dynamics for fruit firmness and softening around an ethylene-dependent polygalacturonase gene in apple (*Malus×domestica* Borkh.). *J Exp Bot* 2010; 61: 3029–3039.

Costa F, Stella S, Van de Weg WE, *et al.* Role of the genes *Md-ACO1* and *Md-ACS1* in ethylene production and shelf life of apple (*Malus domestica* Borkh). *Euphytica* 2005; 141:181-190; DOI 10.1007/s10681-005-6805-4.

Davey MW, Kenis K, Keulemans J. Genetic control of fruit vitamin C contents. *Plant Physiol* 2006; 142: 343–351.

Devoghalaere F, Doucen T, Guitton B, *et al.* A genomics approach to understanding the role of auxin in apple (*Malus x domestica*) fruit size. *BMC Plant Biol* 2012; 12: 7 ; doi: 10.1186/1471-2229-12-7.

Emeriewen OF, Richter K, Hanke MV, [Malnoy](http://link.springer.com/article/10.1007/s10658-014-0565-8#author-details-4) M, Peil A. The fire blight resistance QTL of *Malus fusca* (*Mfu10*) is affected but not broken down by the highly virulent Canadian *Erwinia amylovora* strain E2002A. *Eur J Plant Pathol* 2015; 141: 631; doi:10.1007/s10658-014-0565-8.

# Foster TM, Celton JM, Chagné D, Tustin DS, Gardiner SE. Two quantitative trait loci, *Dw1* and *Dw2*, are primarily responsible for rootstock-induced dwarfing in apple. *Hortic Res* 2015; 2: 15001; doi:10.1038/hortres.2015.1031.

Hemmat M, Weeden NF, Conner PJ, Brown SK, A DNA marker for columnar growth habit in apple contains a simple sequence repeat. *J Am Soc Hort Sci* 1997; 122: 347*-*349.

Kenis K, Keulemans J, Davey MW. Identification and stability of QTLs for fruit quality traits in apple. *Tree Genet. Genomes* 2008; 4: 647–661.

King GJ, Maliepaard C, Lynn JR, *et al*. Quantitative genetic analysis and comparison of physical and sensory descriptors relating to fruit flesh firmness in apple (*Malus pumila* Mill.). *Theor Appl Genet* 2000; 100: 1074–1084.

King GJ, Lynn JR, Dover CJ, Evans KM, Seymour GB. Resolution of quantitative trait loci for mechanical measures accounting for genetic variation in fruit texture of apple (*Malus pumila* Mill.). *Theor Appl Genet* 2001; 102: 1227–1235.

Kumar S, Chagné D, Bink MCAM, *et al.* Genomic selection for fruit quality traits in apple (*Malus* × *domestica* Borkh.). *PLoS ONE* 2012; 7(5):e36674.

Kumar S, Rowan D, Hunt M, *et al.* Genome-wide scans reveal genetic architecture of apple flavour volatiles. *Mol Breeding* 2015; 35: 118; doi:10.1007/s11032-015-0312-7.

Kunihisa M, Moriya S, Abe K, *et al.* Identification of QTLs for fruit quality traits in Japanese apples: QTLs for early ripening are tightly related to preharvest fruit drop. *Breeding Sci* 2014; 64: 240-251.

Liebhard R, Kellerhals M, Pfammatter W, Jertmini M, Gessler C. Mapping quantitative physiological traits in apple *(Malus x domestica* Borkh.). *Plant Mol Biol* 2003a; 52: 511–526.

Longhi S, Moretto M, Viola R, Velasco R, Costa F. Comprehensive QTL mapping survey dissects the complex fruit texture physiology in apple (*Malus x domestica* Borkh.). *J Exp Bot* 2012; 63: 1107–1121.

Longhi S, Cappellin L, Guerra W, Costa F. Validation of a functional molecular marker suitable for marker-assisted breeding for fruit texture in apple (*Malus x domestica* Borkh.). *Mol Breeding* 2013a; 32: 841–852; DOI 10.1007/s11032-013-9912-2.

Longhi S, Hamblin MT, Trainotti L, *et al.* A candidate gene based approach validates Md-PG1 as the main responsible for a QTL impacting fruit texture in apple (*Malus x domestica* Borkh). *BMC Plant Biol* 2013b; 13, 37.

Maliepaard C, Alston FH, van Arkel G, *et al.* Aligning male and female linkage maps of apple (*Malus pumila* Mill.) using multi-allelic markers. *Theor Appl Genet* 1998; 97: 60–73.

Maliepaard C, Sillanpää MJ, van Ooijen JW, Jansen RC, Arjas E. Bayesian versus frequentist analysis of multiple quantitative trait loci with an application to an outbred apple cross. *Theor Appl Genet* 2001; 103: 1243–1253.

Peil A, Flachowsky H, Hanke MV, Richter K, Rode J. Inoculation of *Malus × robusta* 5 progeny with a strain breaking resistance to fire blight reveals a minor QTL on LG5. *Acta Hortic* 2011; 896: 357-362; DOI: 10.17660/ActaHortic.2011.896.49

# Pilcher RL, Celton JM, Gardiner SE, Tustin DS. Genetic markers linked to the dwarfing trait of apple rootstock ‘Malling 9’. J *Am Soc Hortic Sci* 2008; 133: 100–6.

Tartarini S, Gennari F, Pratesi D, Palazzetti C, Sansavini S, *et al.* Characterisation of a race 6 scab resistance gene from Italian germplasm. Acta Hort 2004; 663: 129–133.

Wolters PJ, Schouten HJ, Velasco R, Ammour SI, Baldi P. Evidence for regulation of columnar habit in apple by a putative 2OG-Fe(II) oxygenase. *New Phytol* 2013; 200: 993-999.

Zhu YM and Barritt BH. *Md-ACS 1* and *Md-ACO1* genotyping of apple (*Malus x domestica* Borkh.) breeding parents and suitability for marker-assisted selection. *Tree Genet Genomes* 2008; 4: 555-562.

REFERENCES PEAR

# Knäbel M, Friend AP, Palmer JW *et al.* Genetic control of pear rootstock-induced dwarfing and precocity is linked to a chromosomal region syntenic to the apple **Dw1** loci. *BMC Plant Biol* 2015; 15: 230; DOI: 10.1186/s12870-015-0620-4.

Montanari S, Guérif P, Ravon E, *et al.* Genetic mapping of *Cacopsylla pyri* resistance in an interspecific pear (*Pyrus spp.*) population. *Tree Genet Genomes* 2015; 11: 74; doi:10.1007/s11295-015-0901-y.

Montanari S, Brewer L, Lamberts R, *et al.* Genome mapping of postzygotic hybrid necrosis in an interspecific pear population *Hortic Res* 2016; 3: 15064; doi:10.1038/hortres.2015.64.

# Won K, Bastiaanse H, Kim Y K, *et al*. Genetic mapping of polygenic scab (*Venturia pirina*) resistance in an interspecific pear family. *Mol Breeding* 2014; 34: 2179; doi:10.1007/s11032-014-0172-6.

[Yamamoto](http://www.ncbi.nlm.nih.gov/pubmed/?term=Yamamoto T%5BAuthor%5D&cauthor=true&cauthor_uid=25914590) T, [Terakami](http://www.ncbi.nlm.nih.gov/pubmed/?term=Terakami S%5BAuthor%5D&cauthor=true&cauthor_uid=25914590) S, [Takada](http://www.ncbi.nlm.nih.gov/pubmed/?term=Takada N%5BAuthor%5D&cauthor=true&cauthor_uid=25914590) N, *et al.* Identification of QTLs controlling harvest time and fruit skin color in Japanese pear (*Pyrus pyrifolia* Nakai). [*Breed Sci*](http://www.ncbi.nlm.nih.gov/pmc/articles/PMC4267310/) 2014; 64: 351–361; doi:[10.1270/jsbbs.64.351](http://dx.doi.org/10.1270%2Fjsbbs.64.351).
